# A Review of Antisynthetase Syndrome-Associated Interstitial Lung Disease

**DOI:** 10.3390/ijms25084453

**Published:** 2024-04-18

**Authors:** Puja Patel, Jenna M. Marinock, Aamir Ajmeri, Lawrence H. Brent

**Affiliations:** 1 Section of Rheumatology, Temple University Hospital, Philadelphia, PA 19140, USA; 2 Department of Medicine, Temple University Hospital, Philadelphia, PA 19140, USA; jenna.marinock@tuhs.temple.edu; 3 Department of Thoracic Medicine, Temple University Hospital, Philadelphia, PA 19140, USA; aamir.ajmeri@tuhs.temple.edu

**Keywords:** interstitial lung disease, antisynthetase syndrome, antisynthetase antibodies, idiopathic inflammatory myopathy, anti-Jo-1 antibody, IIM

## Abstract

Our objective in this review article is to present a clinical case of a patient with antisynthetase syndrome (ASyS) and provide an overview of the pathogenesis, classification criteria, antibody profiles, clinical features, and current knowledge of treatment options, focusing on interstitial lung disease (ILD). ASyS is an uncommon autoimmune disease with a heterogenous clinical presentation characterized by the presence of autoantibodies against an aminoacyl-tRNA synthetase and manifested by myositis, fever, inflammatory arthritis, Raynaud’s phenomenon, mechanics hands, and ILD. ASyS-associated ILD (ASyS-ILD) is the most serious complication of ASyS, which may evolve to rapidly progressive ILD; therefore, it often requires thorough clinical and radiologic evaluation including recognition of a specific clinical phenotype associated with the antisynthetase antibodies (ASAbs) to guide therapeutic interventions.

## 1. Introduction

Antisynthetase syndrome (ASyS) is a heterogenous autoimmune disease characterized classically by a triad of muscle inflammation, arthritis, and interstitial lung disease (ILD) [1,2]. It belongs to the family of diseases known as idiopathic inflammatory myopathies (IIMs), which also includes polymyositis, dermatomyositis, immune-mediated necrotizing myopathy (IMNM), inclusion body myositis (IBM), and myositis overlap syndrome [3]. In addition to a broad spectrum of clinical features, ASyS is distinguished by the presence of autoantibodies against aminoacyl tRNA synthetases; however, the role these antisynthetase antibodies (ASAbs) play in the pathogenesis of the disease remains largely unknown, making targeted treatment strategies difficult [4]. This review will serve as a comprehensive discussion of ASyS with an emphasis on ASyS-associated ILD (ASyS-ILD), including updated molecular pathogenic theories, various clinical features, phenotypic profiles, and the latest treatment approaches.

## 2. Clinical Case

A 54-year-old man initially presented to the pulmonary clinic for evaluation of ILD. Six months prior to the current presentation, he had coronavirus disease (COVID-19) with symptoms of low-grade fever, malaise, and a dry cough. Most of his symptoms resolved after one week, aside from the cough and a new inability to take full-deep inspiration. He was treated with an antihistamine, a bronchodilator, and oral corticosteroids. After 1 week, his fever and malaise resolved; however, the cough persisted. He also began experiencing mild dyspnea on exertion. He had no orthopnea, myalgias, rash, or joint pain. The patient revealed a history of Raynaud’s phenomenon.

The patient was born in China and moved to the United States in 1997, where he lived in the northern California, midwestern, and northeastern parts of the country. He had a 10-pack-year smoking history and quit over 30 years ago. The patient did not vape or use alcohol or inhaled or intravenous drugs. Besides having a pet cat, he reported no exposure to other pets or birds, hot tubs or saunas, mold, tuberculosis, or wood or farm work.

In terms of occupation, the patient worked as a scientist in the chemical industry with organic solvent vapor, heavy metal, and dust exposure using a hood. Rarely, he worked with N-methyl-2-pyrrolidine but had no exposure to asbestos, silica, or other metals. The patient also noted working in his home attic with exposed insulation materials containing glass fibers while wearing a simple mask. He reported no use of pneumotoxic medications, herbs, supplements, or radiation therapy.

**Figure 1:**
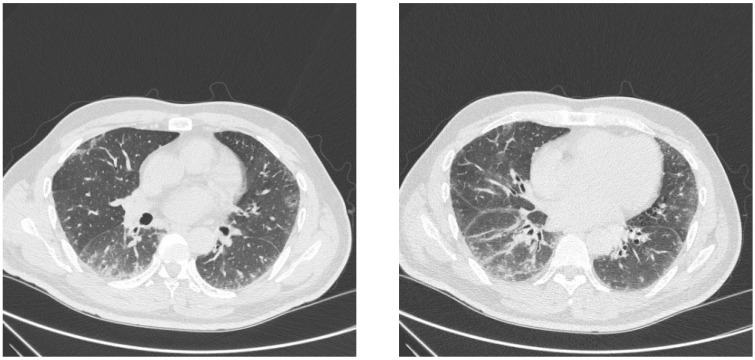
Chest CT scan of lower lung fields.

On physical exam, he was noted to have bibasilar inspiratory crackles and hyperkeratosis of both second digits, right worse than left, and along the right first digit. Pertinent laboratory studies included white blood cell count 13 k/μL (ref 3.8–10.8 k/μL), absolute neutrophil count 10,758 cells/μL (ref 1500–7800 cells/μL), absolute monocyte count 554 cells/μL (ref 200–950 cells/μL), erythrocyte sedimentation rate 34 mm/h (ref ≤ 20 mm/h), C-reactive protein 17.1 mg/L (ref < 8.0 mg/L), creatine kinase 423 U/L (ref 44–196 U/L), antinuclear antibody 1:320 (ref > 1:80 elevated antibody) with cytoplasmic pattern, weakly positive double-stranded DNA antibody 11 IU/mL (ref ≥ 10 positive), and positive EJ 97 SI (ref < 11 SI). The complement C3 and C4 levels were normal. The patient had pulmonary function testing (PFT), which revealed a restriction with reduced total lung capacity (TLC) 4.30/63% predicted, forced expiratory volume (FEV1) prebronchodilator (pre) 1.91/54% and postbronchodilator (post) 2.09/59%, forced vital capacity (FVC) pre 2.34/51% and post 2.58/56%, FEV1/FVC ratio 81, and diffusing capacity of lungs for carbon monoxide (DLCO) 46%. During a 6 min walk test, the patient was able to ambulate 133 m and desaturated to 90% with no significant tachycardia. His computed tomography (CT) scan of the chest demonstrated bilateral lower lobe predominant peripheral and peribronchovascular ground-glass opacities mild traction bronchiectasis (Figure 1).

Given these findings, our patient was diagnosed with connective tissue disease–ILD (CTD-ILD) (amyopathic dermatomyositis/idiopathic inflammatory myopathies spectrum) by pulmonary. He was treated with a prednisone taper and mycophenolate mofetil (MMF) with up titration (500 mg twice daily increased to 1500 mg twice daily). The patient was subsequently referred to rheumatology where his diagnosis was narrowed further to ASyS given the features of Raynaud’s phenomenon, mechanic’s hands, and ILD with positive serology and correlating imaging findings. While on a stable dose of MMF 1500 mg twice daily and tapering his prednisone dose, repeat PFT showed interval improvement with TLC 3.88/61% predicted, FEV1 pre 2.38/77% and post 2.45/79%, FVC pre 2.98/76% and post 2.88/74%, FEV1/FVC ratio 80, and DLCO 64%. On a repeat 6 min walk test, the patient was able to ambulate almost three times as far (365 m) and the lowest oxygen saturation was 92% with mild tachycardia noted. He continued MMF 1500 mg twice daily and the prednisone was tapered off completely with the stabilization of his symptoms and he is currently pending repeat PFT and CT scans.

## 3. Etiology and Pathogenesis

ASyS is defined by the presence of autoantibodies against an aminoacyl-tRNA synthetase. Aminoacyl-tRNA synthetases are a group of twenty cytoplasmic and mitochondrial enzymes, which are essential to RNA transcription and protein synthesis. Autoantibodies against eight aminoacyl-tRNA synthetases have been associated with ASyS, including HisRS (anti-Jo-1), ThrRS (anti-PL-7), AlaRS (anti-PL-12), GlyRS (anti-EJ), IleRS (anti-OJ), AsnRS (anti-KS), PheRS (anti-Zo), and TyrRS (anti-HA/YRS) [5,6,7,8,9,10,11]. Anti-Jo-1 antibodies are most common in patients with ASyS, leaving most of our current knowledge of ASyS focused on this antibody. The clinical associations with different ASAbs are seen in Table 1 and the frequencies of different antibodies in patients with ASyS are seen in Table 2.

HisRS and other aminoacyl-tRNA synthetases are known to be cleaved by granzyme B to produce immunogenic peptides [5,15]. Studies in mice have shown that the N-terminal domain of HisRS can act independently as a chemoattractant for lymphocytes, monocytes, and dendritic cells [16]. Galindo-Feria et al. studied this further by isolating CD4+ T cells from bronchoalveolar lavage (BAL) fluid samples in patients with ASyS, exposing the cells to N-terminal HisRS-derived peptides, and noted the development of a proinflammatory T cell phenotype. The group also isolated germinal center-like structures from lung biopsy samples and anti-Jo-1 antibodies in BAL fluid from ASyS patients. Combined, these results suggest a possible mechanism for ASyS pathogenesis whereby a pulmonary exposure leads to an undefined autoimmune cascade in which HisRS peptide-mediated proinflammatory T cells activate B cells, which in turn form germinal center-like structures that produce anti-Jo-1 antibodies [3,17].

Hervier et al. looked at the involvement of natural killer (NK) cells in the pathogenesis of ASyS by studying the phenotypes of peripheral blood NK cells derived from patients with ASyS vs. healthy controls. They found that ASyS patients had NK cells with a differentiated phenotype and decreased expression of NKp30. This was associated with an overall loss of NK cell function and decreased production of IFN-γ (at baseline and when stimulated). Notably, the NK cells maintain degranulation and proteolytic capabilities when NKp30 expression is decreased. NKp30 expression was not affected by anti-Jo-1 antibodies. Furthermore, in the ASyS group, NK cells were found to be in high quantities in the lungs, with NK cells specifically positive for granzyme B present in both the lungs and in higher quantities in the blood compared to controls [18]. This work taken together with the previous study paints a highly plausible picture for ASyS pathogenesis: a genetically susceptible individual inhales environmental antigens to the exposed lung epithelium where the antigens interact with NK cells, inducing the differentiation and downregulation of NKp30. The production of granzyme B increases and HisRS is cleaved to expose the N-terminal peptide, thus triggering the previously described CD4+ T cell-mediated pathway to produce anti-Jo-1 antibodies [17,18].

Proinflammatory cytokines appear to be involved in the pathogenesis of ASyS. Interleukin-17A (IL-17A) likely plays a major role in the inflammatory process of ASyS. IL-17A, produced by T-helper 17 (Th17) cells, stimulates the production of other proinflammatory cytokines including tumor necrosis factor-alpha (TNF-α), IL-1, IL-6, and others, which promote inflammatory cell recruitment into various tissues [19]. Additionally, IL-17A is involved in the interaction of B and T lymphocytes and in activated B cell immunoglobulin class switching [20]. IL-17A has already been implicated in the pathogenesis of various autoimmune diseases including rheumatoid arthritis (RA) and systemic lupus erythematosus (SLE) where increased serum and tissue levels of IL-17A have been found, and higher serum levels correlated with disease activity in patients [21,22]. Behrens Pinto et al. examined IL-17A serum levels in patients with ASyS and found them to be increased when compared to healthy controls, although the levels did not correlate with disease activity. This research group also conducted a prospective analysis on a subset of these patients who started treatment with rituximab and were followed up at 6- and 12-month intervals. After treatment with rituximab, IL-17A serum levels and disease activity both decreased [19].

The role neutrophils play in the pathogenesis of ASyS has been proposed by the production of neutrophil extracellular traps (NETs) and the process of NETosis. NETosis is the process by which neutrophils release NETs to capture and kill microorganisms in response to infection [23]. Excessive NETosis is hypothesized to play a major role in several autoimmune disorders, as molecules released during NET formation can become autoantigens involved in triggering autoimmune disease [23]. For example, NETs can act as a source of citrullinated proteins, which induce the production of anti-citrullinated protein antibodies (ACPAs) in RA [24]. Inflammatory cytokines, including IL-17A, have been shown to induce NETosis in RA. The increased production of IL-17A in ASyS may also induce NETosis, promoting inflammation and the production of autoantigens [19,24]. Indeed, in IIM studies, NET formation was found to augment the disruption of myofibers, likely through citrullinated histone-mediated pathways [25].

To elucidate the genetic susceptibility to an environmental insult that results in ASyS, López-Mejías et al. looked to a polymorphism of the MUC5B gene (rs35705950), which is associated with idiopathic pulmonary fibrosis (IPF), chronic hypersensitivity pneumonitis (CHP), and RA-ILD. The MUC genes code for mucins, which are part of mucous secretions that are vital to host defense against bacterial and fungal infections, especially in the respiratory tract. The group further conducted an analysis of patients with ASyS, ILD unrelated to ASyS, and healthy controls and found no evidence of a link of *MUC5B* gene polymorphism in patients with ASyS. Likewise, the polymorphism frequency did not correlate with the severity of ASyS-ILD nor with a UIP pattern vs. non-UIP pattern in ASyS-ILD patients. There was, however, a statistically significant increased frequency of *MUC5B* rs35705950 in RA-ILD, IPF, and CHP (which favor a UIP pattern) compared to ASyS-ILD (which favors an NSIP or OP pattern), suggesting these distinct phenotypes result from different genetic predispositions [26].

Remuzgo-Martínez et al., working in the same laboratory, conducted a similar study looking at the *MUC1* rs4072037 allele. This allele was found to increase the risk of ASyS but had no association with the presence or absence of ILD or the anti-Jo-1 status. *MUC1* rs4072037 allele frequencies were higher in ASyS-ILD patients when compared to IPF, although serum KL-6 levels were not statistically different [27]. This suggests that while KL-6 levels cannot be used to differentiate the two diagnoses, the *MUC1* rs4072037 allele could be used as a genetic marker for ASyS-ILD. Ponce-Gallegos et al. studied polymorphisms of the interleukin 1B (*IL1B*), which encodes the proinflammatory cytokine IL-1β, known to be associated with RA, SLE, and other autoimmune disorders. The rs1143634 polymorphism was found to be associated with an increased risk of ASyS and the GA genotype with increased levels of IL-1β in the serum of ASyS patients [28].

## 4. Epidemiology

Although classically presenting with a triad of features including myositis, arthritis, and ILD, ASyS can present with diverse clinical manifestations occurring in various timeframes and thus with a myriad of possible patient presentations. This can be diagnostically challenging. Additionally, the varying classification systems used to diagnose ASyS and various ASAbs make accurate incidence and prevalence data difficult to tabulate. Furthermore, it is likely that patients with ASAbs and myositis are only a portion of patients with ASyS, possibly even a minority, and that there are patients with ILD, arthritis, and other manifestations who are undiagnosed. Even though most of the epidemiological data are from patients with IIM, what is known about ASyS is that women are more often affected than men (greater than 2:1), with a mean age of onset 48 ± 15 years, and African American patients are thought to have more frequent and severe ILD [29,30,31].

## 5. Classification and Diagnostic Criteria

ASyS, while originally categorized as either polymyositis (PM) or dermatomyositis (DM) based on clinical manifestations, is now agreed by most experts to be its own disease entity within IIMs [2,32,33]. However, the latest EULAR/ACR criteria do not yet reflect this, only including anti-Jo-1 and failing to capture ASyS patients positive for one of the seven other ASAbs [34]. In response to this, there have been two major classification criteria proposed for ASyS, Solomon’s criteria and Connors’ criteria, both of which require a positive ASAb as an entry criterion, as seen in Table 3 [30,35].

## 6. Clinical Features

While ASyS is classically defined by a clinical triad including muscle involvement, arthritis, and ILD, patients often do not have all three findings at presentation. Cavagna et al., for instance, found that patients positive for anti-Jo-1 often exhibited an incomplete triad, usually with only one of the manifestations, on presentation. Furthermore, only about half of those patients went on to develop the other two features of the triad later in their disease course, ranging from months to years later [37]. Indeed, many groups have studied the evolution of clinical manifestations, finding various timelines for the development of the triad and other features, including fever, Raynaud’s phenomenon, and mechanic’s hands (Table 4) [38,39,40]. ILD may also be the initial presenting feature, preceding muscle, joint, and cutaneous manifestations.

### 6.1. Muscle

ASyS muscle involvement ranges from myalgias to severe proximal muscle weakness in the upper and lower extremities, resulting in significant disability [32]. Like other types of IIMs, creatine kinase (CK) is often elevated, with one study finding a mean peak CK greater than 4000 UL [32]. About 25% of anti-Jo-1-positive ASyS patients are termed amyopathic, meaning muscle symptoms are absent; however, CK and/or electromyography (EMG) may still be abnormal [37]. Studies have found overall myopathy symptoms are more prevalent and more severe in anti-Jo-1 than anti-PL7 and anti-PL12 ASyS patients (Table 5) [42,43,44]. Magnetic resonance imaging (MRI) and EMG can be useful to examine for the presence of active muscle inflammation and identify target areas for biopsy.

MRI abnormalities were found in 65% of ASyS patients, with nonspecific features including symmetrical muscle loss and edema [45]. EMG findings are indistinguishable from other types of IIMs and include increased spontaneous activity, marked by sharp waves and fibrillation, early recruitment and low-amplitude, and short-duration motor unit potentials [46]. Muscle biopsy, while not always necessary, can help rule out myositis mimics if the diagnosis is unclear. Like dermatomyositis, histology in ASyS patients often reveals perifascicular necrosis [32]. While proximal muscle involvement is most common, esophageal and diaphragmatic weakness can occur, leading to significant dysphagia, gastroesophageal reflux disease (GERD), and dyspnea [32,36].

### 6.2. Joint

Articular manifestations of ASyS typically include polyarthralgias and polyarthritis in roughly 60–70% of ASyS patients [37,39]. When arthritis is present, about 70% have a symmetric polyarticular arthritis resembling RA while 30% have an asymmetric, oligoarticular arthritis [37,39]. Furthermore, of those with an RA-like distribution, it is reported that 35% had erosive changes on plain radiograph, 39% were rheumatoid factor positive, and 28% were anti-citrullinated protein antibodies positive [38]. When symmetric polyarticular arthritis is the sole presenting symptom, a diagnosis of RA is often made until other clinical manifestations evolve over time.

### 6.3. Lung

- Interstitial Lung Disease

Interstitial lung disease is the most common extramuscular manifestation of ASyS with an estimated prevalence of 67–100% [47]. Patients may present with an indolent course of dyspnea on exertion over time, others with a subacute/acute form, and a small portion with a rapidly progressive form leading to high morbidity and potential mortality [12,44]. Still, other patients are discovered to have asymptomatic ILD initially, found only by routine screening. Patients with anti-PL7 or anti-PL12 antibodies were found to have ILD more frequently compared to those with anti-Jo-1 antibodies; furthermore, the patients with anti-PL7 or anti-PL12 tended to have early, severe, and more rapidly progressive ILD [29,44]. Refer to Table 6 for two studies assessing the prevalence of ILD in ASyS by autoantibody.

**Figure 2:**
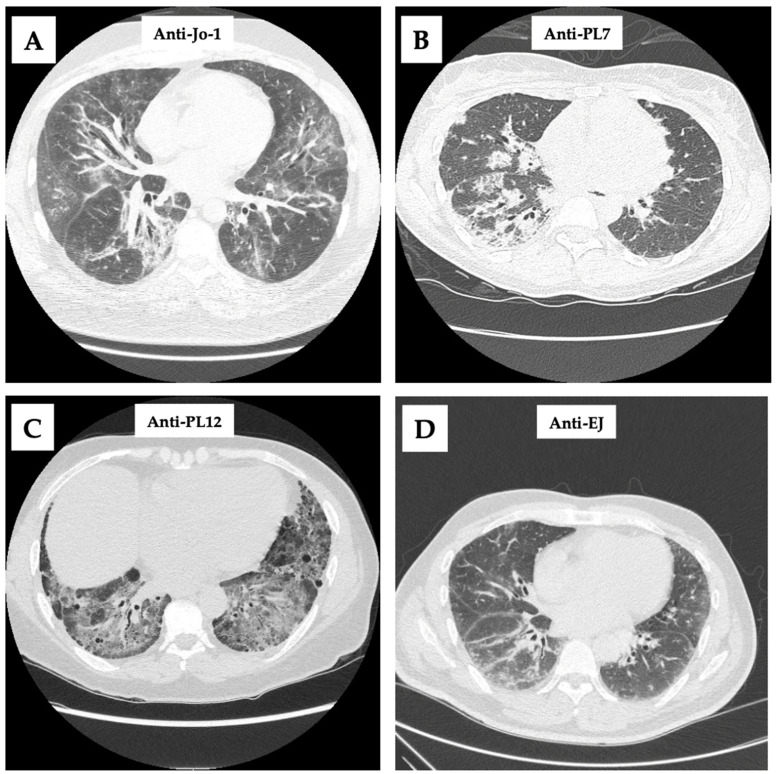
Chest CT scans with different ASAbs. (**A**): Bilateral, scattered, peribronchovascular ground-glass opacities; (**B**): bilateral, right side predominant, dense consolidative opacities; (**C**): diffuse, lower lobe predominant round-glass opacities with traction bronchiectasis; and (**D**): lower lobe predominant, scattered ground-glass opacities.

Fine inspiratory crackles and plain radiographic changes do not occur until late in the course of ILD and should not be relied on for the diagnosis. For baseline screening in asymptomatic patients and for follow-up in patients with dyspnea, pulmonary function testing with DLCO should be performed. A restrictive lung disease pattern with reduced FVC, TLC, and DLCO is suggestive of ILD and should be followed with high-resolution computed tomography (HRCT) of the chest, the gold-standard for diagnosis [36]. HRCT is essential to the diagnosis of ASyS-ILD and especially helpful when ILD is the lone presenting manifestation. Several studies found that 78–100% of patients with ASyS-ILD on HRCT have nonspecific interstitial pneumonia (NSIP), organizing pneumonia (OP), or a mixed NSIP/OP pattern [36,49,50,51,52,53]. When these patterns are found in a patient with isolated pulmonary symptoms, it should prompt the physician to look for ASyS-specific autoantibodies. Refer to Figure 2 for radiographic findings of various ILD patterns based on different ASAbs.

- Pulmonary Arterial Hypertension

Like other autoimmune conditions, pulmonary arterial hypertension (PAH) has been shown to occur in patients with ASyS, both secondary to and independently from ILD [54]. One retrospective study by Hervier et al. found that, using transthoracic echocardiograms (TTEs), 25% of their cohort had probable PAH, with 7.9% confirmed to have severe pre-capillary PAH by right heart catheterization (RHC). As only a few selected people underwent RHC, this likely underestimates the number with severe PAH [55]. The presence of PAH was associated with significant mortality with a reported three-year survival rate of 58% [55,56]. Due to the poor prognosis associated with PAH, asymptomatic patients should undergo routine screening with TTEs.

### 6.4. Skin

- Mechanic’s Hands and Hiker’s Feet

**Figure 3:**
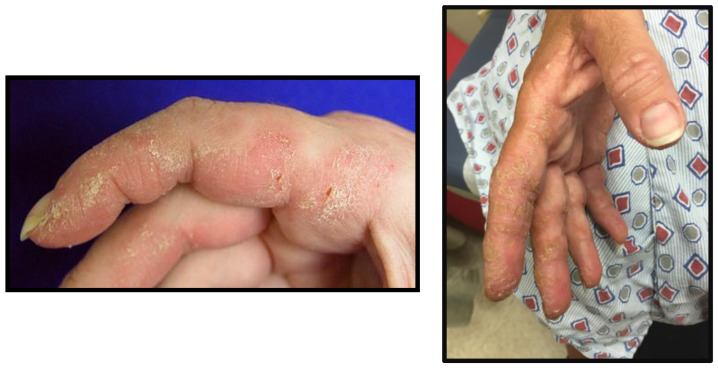
Mechanic’s hands (courtesy of C. Oddis).

Mechanic’s hands are a classic cutaneous finding in ASyS marked by scaly, fissured, hyperkeratotic plaques on the radial and palmar aspects of the fingers and hands, especially the second finger [57]. The lesions are named for resembling an occupational dermatitis associated with manual labor, as seen in Figure 3. Mechanic’s hands, however, are not specific to ASyS and can occur in other types of myositis. Similar eruptions have been noted on the toes of patients with ASyS and termed “hiker’s feet” in recent years [58,59]. Hiker’s feet were noted to occur in 90% of patients who had mechanic’s hands [58]. Notably, some report an association between the presence of mechanic’s hands and a higher rate of ILD [57].

- Raynaud’s Phenomenon, Nailfold Capillary Abnormalities, and Digital Ischemia

ASyS has several clinical features that overlap with those of systemic sclerosis (SSc), including Raynaud’s phenomenon, nailfold capillary abnormalities, and digital ischemia [12]. Raynaud’s phenomenon (RP) is thought to occur in 38–51% of patients with ASyS [3]. Nailfold capillary abnormalities have been reported in over 60% of ASyS patients, with 35% having an SSc-like pattern and occurring independent of RP [60]. Digital ischemia was previously thought to occur infrequently in ASyS but may be underestimated due to misclassification as SSc. A single-center cohort in Japan was found to have severe digital ischemia in 7% of their patients positive for ASAb [61].

### 6.5. Other Clinical Features

While other types of IIMs, especially dermatomyositis, are associated with malignancy, there are mixed data as to whether ASyS poses an increased risk of malignancy or is actually protective compared to other types of IIMs [29]. However, given ASyS patients are often treated with long-term immunosuppressants, routine cancer screenings are still essential. Besides PAH, other cardiac manifestations of ASyS may include myocarditis or pericarditis, although they are typically of minor clinical significance [62,63].

## 7. Imaging and Histology

HRCT of the chest is the gold-standard imaging for detecting ASyS-ILD, revealing unique imaging patterns compared to other causes of ILD. Waseda et al. described these features as ground-glass opacities, consolidation, and reticulation, distributed in the lower lobes, periphery, and/or peribronchovascular regions, most consistent with NSIP. The NSIP pattern alone was found in >55% of ASyS-ILD cases. The remaining cases consisted of an OP pattern (marked by patchy areas of peripheral, subpleural, and peribronchiolar consolidations) or a mixed NSIP/OP pattern [50]. The usual interstitial pneumonia (UIP) pattern (marked by honeycombing and traction bronchiectasis) has also been described but is less frequent than NSIP [49,50,64]. Diffuse alveolar damage (DAD) was found mostly in patients with anti-Jo-1. A separate case series of 22 ASyS-ILD patients similarly had a predominance of NSIP and OP patterns; however, they also had a high rate of acute presentations with nearly 20% of patients displaying acute interstitial pneumonia on HRCT [65]. The radiographic pattern itself is less important than recognizing that a variety of ILD patterns may ultimately be due to ASyS-ILD. Recent case reports have highlighted this as well, as patients initially suspected to have COVID-19 pneumonia were later diagnosed with ASyS-ILD based on CT imaging patterns and subsequent serology testing [66,67]. Refer to Table 7 for the frequency of ILD patterns associated with various ASAbs.

Lung biopsies for histopathological confirmation of HRCT findings are not routinely performed in ASyS-ILD. For one, patients with a rapidly progressive ILD often have minimum pulmonary reserve to undergo an invasive procedure. Secondly, biopsies often have low diagnostic utility, other than to rule out infectious etiologies, as diagnosis is centered around clinical, serological, and HRCT findings. Furthermore, there are no known pathological findings specific to ASyS-ILD [64]. Finally, surgical lung biopsies are associated with significant morbidity and may lead to a flare of the underlying lung disease [70].

Chartrand et al. used a retrospective approach to study 33 patients thought to have idiopathic interstitial pneumonia before being ultimately diagnosed with ASyS by serology plus HRCT and/or surgical lung biopsy. On HRCT, 31/33 (94%) patients were found to have an NSIP, an OP, or a mixed NSIP/OP pattern. Surgical lung biopsy was performed on 21 patients, with 19 (90.5%) having an NSIP, OP, NSIP/OP, NSIP/DAD, or OP/DAD pattern. Additional histopathologic features suggesting an underlying autoimmune etiology were noted, including lymphoplasmacytic infiltration, lymphoid hyperplasia +/− germinal centers, and evidence of pleuritis or inflammatory airways disease [53]. These findings reinforce the notion that histopathological results may not differ substantially from skilled HRCT image interpretation but are another investigative tool available in case of diagnostic uncertainty.

Muscle biopsies are easier to obtain and used more frequently to study IIM, with more recent research focused on identifying specific pathological findings unique to ASyS. Noguchi et al. looked at 50 patients with ASyS and found a pattern of perifascicular necrosis in 48% of samples and diffuse myofiber necrosis in 16%. The remaining 36% of samples revealed few necrotic fibers, perimysial mononuclear cellular infiltration, and/or MHC class I and II expression on the sarcolemma and cytoplasm of non-necrotic myofibers [32]. Work by da Silva et al. using muscle biopsy samples from 26 anti-Jo-1-positive ASyS patients with objective muscle involvement found nearly 40% of the patients had a necrotizing myopathy pattern on histological review, marked by the presence of CD68+, and low CD4+ and CD8+ staining [71]. These necrotizing myopathy patterns described are more classically seen in IMNM; however, taken in the right clinical context, they may support a diagnosis of ASyS. As a non-Jo-1 ASAb can be more technically challenging to isolate, Tanboon et al. sought to explore the diagnostic utility of HLA-DR myofiber expression in ASyS. The group compared HLA-DR staining patterns from muscle biopsies of ASyS and non-ASyS myositis samples and additionally performed RNA sequencing to evaluate interferon (IFN)-signaling pathway genes [72]. Both HLA-DR expression and IFN-y-related gene upregulation were prominent in ASyS and IBM, suggesting HLA-DR expression could further support ASyS diagnosis in the right clinicopathological context.

## 8. Autoantibodies Phenotypic Profiles

To date, eight ASAb have been associated with ASyS, including anti-Jo-1 (anti-histidyl tRNA synthetase), anti-PL7 (anti-threonyl tRNA synthetase), anti-PL12 (anti-alanyl tRNA synthetase), anti-OJ (anti-isoleucyl tRNA synthetase), anti-EJ (anti-glycyl tRNA synthetase), anti-KS (anti-asparaginyl tRNA synthetase or AsnRS), anti-HA/YRS (anti-tyrosyl tRNA synthetase or TyrRS), and anti-Zo (anti-phenylalanyl tRNA synthetase or PheRS) [5,6,7,8,9,10,11]. It is extremely rare for patients to have more than one ASAb and it is thought that different phenotypic profiles specific to each ASAb exist (see Table 8). Along with ASAbs, other autoantibodies including anti-citrullinated peptide/protein antibodies (ACPAs) and anti-Ro52 have been associated with distinct ASyS phenotypes.

- Anti-Jo-1 (Anti-Histidyl tRNA Synthetase)

Anti-Jo-1 is the most common ASAb, occurring in roughly 30–60% of ASyS patients depending on the cohort studied [47,49,73,74]. It is associated more frequently with polyarticular arthritis and muscle involvement compared to the other ASAbs [47,56,75]. Indeed, an isolated arthritis at disease onset was the most common presentation for anti-Jo-1 [14]. Mechanic’s hands were more common in an anti-Jo-1 cohort, as well as lower CK serum values [76]. ILD, when present, often has an NSIP pattern on HRCT and is less severe in ASyS patients positive for anti-Jo-1 [29,44,69]. One study suggested that higher levels of anti-Jo-1 antibodies correlated with disease activity and severity [77]. Across several cohorts, a positive anti-Jo-1 antibody coincided with improved overall survival [29,56,74]. In summary, anti-Jo-1 tends to produce a milder phenotype of ASyS with a favorable disease course and prognosis.

- Anti-PL7 (Anti-Threonyl tRNA Synthetase) and Anti-PL12 (Anti-Alanyl tRNA Synthetase)

Anti-PL7 and anti-PL12 ASAbs are often studied together as they display a similar phenotypic profile, including more prevalent and severe ILD compared to anti-Jo-1 [29]. In fact, isolated ILD is the most common presenting symptom at disease onset for anti-PL7/PL12 [14,78]. Based on two separate retrospective cohort studies, anti-PL12 and anti-PL7 were often associated with NSIP patterns on HRCT and/or transbronchial biopsy; however, anti-PL12 was also linked to an OP pattern and anti-PL7 with a UIP pattern [69,79].

Myositis occurs in about 50–75% of anti-PL7/PL12-positive patients, who also have higher rates of GERD and dysphagia [29,44,63,78,80]. The gastrointestinal (GI) manifestations may be secondary to myositis or to intrathoracic pressure changes in the setting of underlying ILD but need to be further studied [29]. Of interest, some retrospective studies found increased cardiovascular events including pericarditis, myocarditis, and PAH in anti-PL7/PL12 ASyS patients [63,80]. Those positive for anti-PL7 had especially high rates of pericarditis, occurring in one-third to one-half of patients [63,78]. Anti-PL7/PL12 positivity was further associated with a greater diagnostic delay and lower survival rates compared to anti-Jo-1 [14,74].

- Anti-OJ (anti-isoleucyl tRNA synthetase)

Of the commercially available myositis panels, anti-OJ is the least commonly detected ASAb [36]. One large retrospective study found that at the time of diagnosis, ASyS patients with anti-OJ most often had isolated myositis, while another cited isolated ILD [14,81]. The myositis tends to be particularly severe, with higher CK values, more necrosis on muscle biopsy, and profound weakness when compared to the myositis course associated with other ASAbs [32,81]. This was also confirmed via muscle biopsy, in which anti-OJ samples showed myopathological features including diffuse myofiber necrosis, which corresponded clinically to severe myositis compared to other ASAbs [32,72]. Depending on the cohort studied, anti-OJ-positive ASyS-ILD has been associated with NSIP/OP or UIP patterns on HRCT [69,79].

- Anti-EJ (anti-glycyl tRNA synthetase)

As seen in anti-PL7/PL12, isolated ILD at disease onset is the most frequent presentation for anti-EJ [14]. Anti-EJ-positive ASyS-ILD is also associated with the most common HRCT patterns: NSIP or OP [69,79]. Furthermore, ILD is responsive to immunotherapy, but recurrences are common [82].

- Anti-KS (anti-asparaginyl tRNA synthetase), Anti-HA/YRS (anti-tyrosyl tRNA synthetase), Anti-Zo (anti-phenylalanyl tRNA synthetase)

Limited data exist for anti-KS, anti-HA/YRS, and anti-Zo ASyS as these antibodies must be detected via immunoprecipitation, which is not widely available commercially. Anti-KS most often presents with isolated ILD, in more than three-quarters of ASyS patients positive for the antibody from one Japanese retrospective study [73]. When looking at ASyS-ILD, anti-KS positivity was associated more often with NSIP or UIP patterns on imaging [83,84]. A small case series in the UK of patients with anti-Zo ASyS showed similar clinical features as the other ASAbs previously described [85].

- ACPA (anti-citrullinated peptide/protein antibody)

ACPA, best known for high specificity for RA, has been found in the serum of patients with other autoimmune conditions, such as SLE and SSc, and often coincides with an RA-like erosive arthritis [86,87,88,89,90]. Meyer et al. used a case–control study to evaluate the clinical significance of the ACPA in ASyS patients. The group found all ASyS patients with ACPA positivity had an RA-like polyarthritis involving small joints, positive rheumatoid factor, and elevated C-reactive protein; thus, while patients met the ASyS criteria, they also met the diagnostic criteria for RA. This can often lead to diagnostic uncertainty and the delayed diagnosis of ASyS in patients with isolated arthritis. Furthermore, Meyer et al. found that ACPA-positive ASyS was also associated with refractory arthritis despite the increased use of disease-modifying antirheumatic drugs (DMARDs) and an overall higher incidence of joint damage. Notably, a small subset of patients treated with a TNF-α inhibitor developed worsening ILD and/or myositis, suggesting that rituximab may be a more suitable treatment in ACPA-positive ASyS patients [91].

- Anti-Ro52

The Ro/SSA antigen has two polypeptide components Ro52 and Ro60, both commonly implicated in other autoimmune diseases; however, only antibodies against Ro52 are considered to be myositis-associated [92]. Notably, it was shown to be an independent predictor for the development of ILD [93,94,95]. In IIM patients, anti-Ro52 is strongly associated with ASyS, ILD, and dysphagia [96]. One prospective observational study found patients with isolated anti-Ro52 antibodies and non-rapidly progressive ILD to have an NSIP pattern on HRCT, whereas those who developed rapidly progressive ILD more often had an OP pattern [94].

Anti-Ro52 frequently occurs with ASAbs, most commonly with anti-Jo-1 in Caucasian-predominant cohorts and with anti-PL7/PL12/EJ in Southeast Asian-predominant cohorts [41,74,97,98,99]. Anti-Ro52 is associated with an increased frequency of ILD and, in one ASyS cohort, with a decreased frequency of muscle involvement and strong female prevalence [100]. In several retrospective studies, coincident anti-Ro52 antibodies were associated with more severe ILD, often refractory to many treatment options, and associated with a poor overall prognosis [43,92,97,101,102]. One group found that the response to immunosuppressive therapy was dependent on the concentration of anti-Ro52 in the serum, aside from rituximab which worked well regardless of the anti-Ro52 presence or concentration [92]. It therefore has been proposed to screen ASyS patients for anti-Ro52 for risk stratification and preferred treatment options.

**Table 8:** Phenotypes of ASyS by ASAb specificities.

Predominant Feature		Antisynthetase Antibodies	Common Phenotype
Arthritis Myositis ILD	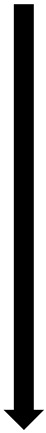	Anti-Jo-1	Isolated arthritis common [14] Mild myositis [76] ILD with NSIP pattern; overall, less severe with improved overall survival [29,44,69] Mechanic’s hands common [76]
		Anti-OJ	Often isolated myositis or ILD [43,81] Myositis tends to be severe [32,81] ILD with NSIP, OP, or UIP [69,79]
		Anti-PL7	More severe ILD with NSIP or UIP pattern [69,79] Myositis common [29,44] More frequent GERD and dysphagia [29]
		Anti-PL12	More severe ILD with NSIP or OP pattern [69,79] Myositis common [29,44] More frequent GERD and dysphagia [29]
		Anti-EJ	Isolated ILD with NSIP or OP pattern [69,79]
		Anti-KS	Isolated ILD with NSIP or UIP pattern [83,84]
		Anti-HA/YRS and Anti-Zo	Not enough information is known

## 9. Treatment of ILD in ASyS

The treatment and management of ASyS, with its heterogeneous multisystem manifestations, is often challenging and requires a collaborative multidisciplinary approach including the rheumatologist, pulmonologist, dermatologist, and radiologist. There are no FDA-approved medications for the treatment of ASyS and the treatment approach is usually targeted at the organ systems involved and the disease severity. The general treatment paradigm for ILD in ASyS is extrapolated from studies of patients with myositis associated with ILD. Usually, the management of ILD in ASyS is targeted at both pharmacologic and non-pharmacologic strategies as well as disease monitoring. However, in this review, we will focus the discussion on pharmacologic interventions in the management of ILD in ASyS. Table 9 summarizes the American College of Rheumatology (ACR) guidelines for the treatment of ILD in systemic autoimmune rheumatic diseases.

### 9.1. Glucocorticoids

Glucocorticoids (GCs) have long been the initial mainstay treatment for ILD in ASyS. There is a lack of consensus or guidelines recommending a standard regimen for the initial dose, duration, or tapering strategies for GCs. However, there is strong evidence suggesting clinical recurrence of lung disease with GC tapering [103] and often glucocorticoid-sparing immunosuppressive agents are needed to manage the disease. The initial dose selected is based on the acuity, the severity of the symptoms, and the degree of respiratory impairment. Usually, patients are started on 1 mg/kg per day of prednisone (or equivalent and no more than 60–80 mg daily) or pulse therapy of methylprednisolone 1000 mg for 3 days for rapidly progressing ILD. Other immunosuppressive agents are often added to glucocorticoid therapy to facilitate tapering and to minimize adverse effects associated with the use of glucocorticoids, which is dependent on the dose and the duration of therapy. Common adverse effects are weight gain, insomnia, accelerated decrease in bone mineral disease, high blood pressure, hyperglycemia, and cushingoid features, but any organ system can be affected.

### 9.2. Mycophenolate Mofetil

Mycophenolate mofetil (MMF) (or myfortic acid) is a widely used immunosuppressive agent for the treatment of various manifestations of several rheumatological conditions, including SLE, myositis, systemic vasculitis, SSc, and inflammatory eye disorders. MMF is converted to mycophenolic acid, which is an active metabolite that inhibits inosine monophosphate dehydrogenase, leading to suppressed T and B cell lymphocyte proliferation.

In several case reports and retrospective case series of patients with IIM-associated ILD (IIM-ILD) and other CTD-ILD, MMF demonstrated the improvement and stabilization of declining lung function [104,105,106,107,108]. Typically, MMF is initiated at a dose of 500 mg twice a day, with a target dose of 2–3 g per day. GI symptoms are the most commonly noted adverse effect and can be curtailed with the use of enteric-coated mycophenolic acid. Other observed adverse effects include cytopenias due to bone marrow suppression, infections, and possibly increased risk of malignancy.

### 9.3. Azathioprine

Azathioprine (AZA) is another commonly used immunosuppressive agent that is used for the treatment of a variety of conditions, including numerous rheumatic diseases. AZA is a prodrug, which is converted to its active metabolite 6-mercaptopurine that inhibits DNA replication by inhibiting purine synthesis. Similar to MMF, data for AZA use in ASyS-ILD are derived from studies of patients with IIM and IIM-ILD. There is little evidence in the literature comparing the efficacy of AZA to other immunosuppressive agents for use in patients with ASyS-ILD. A retrospective study of 46 patients (positive Anti-Jo 1 antibody in 50%) showed the stabilization of lung function and a reduction in steroid dose in 24 who were treated with cyclophosphamide (CYC), 12 with AZA, and 9 with MMF [109]. In a single-center retrospective observational study of 110 patients with IIM-ILD, 66 were treated with AZA and 44 with MMF. This study adjusted for ASAbs but showed that the use of both AZA and MMF was associated with an improved FVC % predicted and a lower prednisone dose. AZA was also associated with an improvement in the DLCO % predicted and MMF showed stabilization of the DLCO % predicted in patients with IIM-ILD over 2 to 5 years. In this cohort, a higher rate of adverse events was noted more frequently in the AZA group than the MMF group, 17% vs. 7.5%, respectively [110]. 

Prior to the initiation of AZA, it is imperative that patients are tested for thiopurine methyltransferase (TMPT) deficiency to guide the dose adjustment in poor metabolizers. AZA is usually started at 50 mg per day, with a goal target dose of 2–3 mg/kg per day to minimize adverse events. The most common adverse effects of AZA include GI disturbances, bone marrow suppression, risk of infection, and possibly malignancies. 

### 9.4. Calcineurin Inhibitors

Calcineurin inhibitors (CNIs) such as cyclosporine and tacrolimus exert their immunosuppressive effects through T cell inhibition. They are routinely used to prevent the rejection of solid organ transplantation but are occasionally used as an adjunctive treatment in autoimmune diseases. In a 2015 systematic review assessing the efficacy of tacrolimus in patients with refractory IIM-ILD, an improvement in or stabilization of FVC in 89.3% (25/28) and DLCO in 81.3% (13/16) was noted. The ASyS-ILD patients with the anti-Jo-1 or anti-PL-12 antibodies showed stabilization of 56.6% (30/53) and 3.8% (2/53), respectively [111]. In a retrospective Japanese study of 32 patients with ASyS-ILD treated with GC monotherapy (n = 12) vs. GC plus CNI (n = 20) showed in the entire cohort a 2-year progression-free survival and 5-year survival rate of 68.8% and 96.9%, respectively. Although, there was no significant difference regarding long-term survival between the two groups [112]. 

Typically, tacrolimus is started at 1 mg twice daily, which is subsequently titrated based on target trough levels of 3–6 ng/mL. Renal dysfunction is the most common and significant side effect; therefore, it is imperative that serum trough levels of tacrolimus are closely monitored, especially in patients with fluctuating renal clearance. Other potential adverse events include hypertension, electrolyte abnormalities, headache, infections, and increased risk of malignances.

### 9.5. Rituximab

Rituximab (RTX) is a chimeric monoclonal antibody directed against the CD20 antigen on the surface of B lymphocytes, resulting in B cell depletion. It was the first monoclonal antibody approved for the treatment of cancer, specifically hematologic B cell malignancies. Since then, numerous studies have shown the efficacy of B cell depletion in autoimmune diseases, leading to FDA approval for use in rheumatoid arthritis and ANCA-associated vasculitis, as well as off-label use in several other rheumatic diseases [113]. The use and benefit of rituximab has been demonstrated in several retrospective series of patients with progressive and treatment-refractory ASyS-ILD, by showing improvement in the PFT and HRCT findings [114,115,116,117]. An open-label, phase II clinical trial (n = 10) assessing the efficacy of rituximab in patients with ASyS-ILD refractory to multiple immunosuppressive agents showed improvement in PFT (measured by an increase of 10% in the absolute FVC or 15% in the DLCO) for 5 patients, stabilization in 4 patients, and worsening in 1 patient [118]. The authors of this study noted that only two patients had an improvement in the DLCO, suggesting that the improvement in PFT could be due to an improvement in muscle strength rather than an improvement in ILD. Nonetheless, this study with a relatively small sample size emphasized the need for large clinical trials.

Adverse events include infusion reactions to rituximab, which are usually mild but rarely can be life-threatening. However, serious toxicities include infections, hypogammaglobulinemia, viral reactivation (hepatitis B), and cytopenias. Typically, two doses of intravenous rituximab of 1000 mg are administered two weeks apart every 6 months, but the dosing regimen has not been standardized.

### 9.6. Cyclophosphamide

Cyclophosphamide (CYC) is an alkylating agent that is used to treat severe organ-threatening manifestations of a variety of rheumatic diseases. CYC is a prodrug that is converted to active metabolites in the liver that exhibits cytotoxic effects by crosslinking strands of DNA and RNA. It is a potent and effective immunosuppressive agent but has the potential for severe toxicity in the short and long term. Its efficacy was demonstrated in a 2015 systematic review that assessed five studies of IIM-ILD patients (>50% patients positive for anti-Jo-1) treated with intravenous (IV) CYC. The main outcome (an increase of ≥10% in vital capacity (VC) and DLCO or decreases of ≥10% in HRCT score) was noted in 57.6% (34/59), 64.3% (27/42), and 67.3% (35/52) of patients with improvements in VC, DLCO, and HRCT, respectively [119]. Another systematic review and meta-analysis published in 2019 that included 553 patients (45% with ASyS) showed an improvement in lung function by 56.4% with CYC (95% CI 44–68.0) versus by 89.2% with GC monotherapy (95% CI 82.5–93.6). However, in rapidly progressive ILD, the survival rates at 3 months were 72.4% (95% CI 6.4–99.0) for CYC versus 51.7% (95% CI 24.2–78.1) for GC alone [120]. A recent observational comparative study of patients with IIM-ILD (31 with ASyS) showed an FVC improvement in 64% of patients in the CYC groups versus 32% of patients in the non-CYC group; however, patients in the CYC group received more methylprednisolone pulses (59% vs. 28% in the non-CYC group), lower initial GCs doses >30 mg/d (19% vs. 77%), and lower 6-month average doses of prednisone (11 mg/d vs. 31.1 mg/d) [121]. Notably, this study excluded patients with a UIP pattern and anti-MDA-5 positive myositis, which have both been associated with the rapid deterioration of ILD [122,123]. 

Several studies of patients with IIM-ILD including ASyS have compared the efficacy of RTX vs. CYC. One retrospective observational study of 62 patients with ASyS-ILD showed that RTX was associated with a better 2-year pulmonary progression-free survival compared to CYC, despite similar progression-free survival at 6 months [124]. Most recently, in 2023, a first double-blind, double-dummy, randomized controlled phase 2b (RECITAL) trial assessed the efficacy of RTX versus IV CYC in patients with severe or progressive CTD-ILD. Of the 91 participants, 44 (45%) had IIM-ILD, and at 24 weeks, the FVC improved from baseline in both groups, showing no benefit of RTX over CYC. However, the overall incidence of adverse events was more commonly reported in the CYC group (646 events) versus the RTX group (445 events), including more serious adverse events in the CYC group (62) versus RTX group (33) [125]. Even though this study did not include the antibody profile of patients with IIM-ILD, it serves as a practical guide for clinicians. 

Although CYC is very effective, its use is limited to severe cases of ILD due to issues related to toxicity, including its effects on the bone marrow, reproductive organs, and bladder. Therefore, only clinicians familiar with the pharmacology, administration, and monitoring should prescribe CYC. A thorough pre-treatment evaluation should be completed prior to administration, including baseline laboratory tests, urinalysis, screening for latent tuberculosis, and hepatitis B and C serologies.

### 9.7. CD19-Targeted Chimeric Antigen Receptor T Cells

Treatment with chimeric antigen receptor (CAR) T cells has emerged as an effective therapy in patients with hematological malignancies, such as B cell lymphoma, acute lymphoblastic leukemia, and multiple myeloma. In recent years, treatment with autologous CAR T cells directed against the B cell-specific surface molecule CD19 have shown rapid clinical remission and efficacy in several treatment-refractory rheumatic conditions, including SLE [126,127,128] and SSc [129]. Similarly, the use of CD19 targeting CAR T cells against B cells and plasmablasts has shown complete remission in patients with the Jo-1 antibody refractory to several immunosuppressive therapies, including RTX [130,131,132]. Cytokine release syndrome is observed in all patients, which can range from mild to life-threatening, especially in patients with hematological malignancies. However, a retrospective review of cases showed that patients with autoimmune conditions treated with CAR-T cell therapy exhibit less toxicities and serious adverse effects than in patients with B cell lymphoma [133]. While the results are encouraging, this novel therapy is costly and not widely available and limited follow-up studies exist. 

### 9.8. Other Biological Agents

A systematic literature review and case series demonstrated a beneficial role of IL-1 inhibition with anakinra in seven patients with difficult to treat manifestations of ASyS, despite the use of other immunosuppressive agents [134]. Similarly, IL-6 blockade with tocilizumab in two patients with treatment-refractory ASyS-ILD showed an improvement in and stabilization of lung function over time [135]. Abatacept (CTLA4-Ig) used in eight patients with ASyS-ILD refractory to other therapeutic agents was well tolerated and showed improvement in FVC and DLCO [136]. A randomized controlled pilot trial (ClinicalTrials.gov Identifier: NCT03215927) to evaluate the efficacy and safety of abatacept in treating ASyS-ILD was completed in July, 2023, but the results are not published yet. Several case reports have also demonstrated the efficacy of Janus kinase (JAK) inhibitors in treatment-refractory ASyS-ILD [137,138,139]. 

### 9.9. Intravenous Immunoglobulin

Intravenous immunoglobulin (IVIG) is used in a variety of autoimmune and inflammatory disorders [140]. IVIG is FDA-approved for the treatment of dermatomyositis [141]. A retrospective study of 19 patients with active progressive ASyS-ILD treated with IVIG showed an increase in the mean percent-predicted FVC and (*p* = 0.048) and percent-predicted DLCO (*p* = 0.0223). Seven patients had a >10% increase in FVC (five patients at 6 months, one patient at 12 months, and one patient at 24 months) and five patients showed a >10% or greater improvement in DLCO and total lung capacity over time. Despite demonstrating improved lung function, about 53% of patients developed mild-to-severe adverse effects [142].

#### 9.9.1. Therapeutic Apheresis

Apheresis involves the extracorporeal removal, return, or exchange of large substances from the blood or plasma. Two case reports have demonstrated its use in ASyS refractory to steroids and other therapeutic agents [143,144]. 

#### 9.9.2. Antifibrotic Agents

Antifibrotic agents, pirfenidone and nintedanib, were approved in 2014 by the FDA for the treatment of idiopathic pulmonary fibrosis. Although antifibrotic agents have been proven to be safe and efficacious for the treatment of chronic progressive fibrosing ILD [145,146,147], especially SSc-ILD [148], there is a lack of large, randomized trials that assess the efficacy of antifibrotic agents for the treatment ASyS-ILD or IIM-ILD. A retrospective multicenter pilot study comparing 36 patients receiving nintedanib and 115 patients not receiving nintedanib showed improved survival (HR: 0.26 and 95% CI: 0.09–0.75) and a lower incidence of rapidly progressive ILD (OR: 0.09 and 95% CI: 0.16–0.55) in the nintedanib group [149]. A retrospective cohort study in China of 307 patients with interstitial pneumonia and myositis-specific antibodies showed that the addition of antifibrotic agents in combination with glucocorticoids and immunosuppressive agents showed a reduced risk of developing fibrosis at a median follow-up period of 22 months (HR 0.32, 95% CI 0.11–0.90, and *p* = 0.03) [150].

Typically, pirfenidone is administered with food in three divided doses, which ranges up to 40 mg/kg per day (not to exceed 2403 mg/day). Common adverse effects include rash, photosensitivity, GI manifestations, fatigue, and anorexia. Although rare, severe cutaneous adverse reactions such as a drug reaction with eosinophilia and systemic symptoms, Stevens–Johnsons syndrome, and toxic epidermal necrolysis have been reported. Lastly, nintedanib is initiated at a standard dose of 150 mg twice daily. GI discomfort, diarrhea, and liver function test abnormalities are the most common side effects of nintedanib. Its use is not recommended in patients with severe hepatic impairment and patients taking full anticoagulation due to its interaction with CYP3A4 inhibitors and inducers.

## 10. Conclusions

ASyS is a complex multisystem disease with a heterogenous presentation and clinical features. Because patients with ASyS have other clinical features other than myositis as a presenting manifestation, many patients are undiagnosed. This was evident in our patient who presented with an insidious onset of subtle respiratory symptoms, a history of previous COVID-19 infection, and significant occupational exposures, all making for a diagnostically challenging case. Through keen observation and physical examination, the finding of mechanic’s hands was discovered, prompting further investigation, which included serological testing and imaging modalities. This subsequently led to the diagnosis of ASyS, emphasizing the importance of a multidisciplinary collaborative approach needed for the optimal management of ASyS-ILD. Prompt recognition and treatment is imperative as ASyS is associated with a high prevalence and an increased severity of ILD compared with other IIMs, often leading to high morbidity and mortality in these patients. Our patient was treated with systemic steroids and MMF with a subjective alleviation of respiratory symptoms and had improvement in PFT. Although evidence guiding treatment decisions is limited and mainly supported by case series and retrospective studies, timely recognition is of utmost importance to allow for early treatment. Further research and prospective studies with only a cohort of ASyS patients are needed to assess the efficacy of treatments, as current therapeutic approaches are extrapolated from patients with IIM-ILD.

## Figures and Tables

**Table 1:** Frequency of various ASAbs in patients with IIM and associated clinical features; Huang, 2020 [12].

Antibody	Prevalence in Patients with Myositis	Myositis	Arthritis	ILD
Anti-Jo-1	15–30%	82%	74%	82%
Anti-Pl-7	2–5%	80%	50%	77%
Anti-PL-12	2–5%	51%	42%	83%
Anti-EJ	<2%	84%	53%	90%
Anti-OJ	<2%	78%	44%	61%
Anti-KS	<2%	-	-	-
Anti-Zo	<1%	-	-	-
Anti-Ha	<1%	-	-	-

**Table 2:** Prevalence of ASAbs in patients with ASyS.

Antibody	Zhao, 2022 [13] n = 111	Cavagna, 2019 [14] n = 828
Anti-Jo-1	63%	72%
Anti-Pl-7	15%	11%
Anti-PL-12	8%	10%
Anti-EJ	14%	5%
Anti-OJ	-	2%
Anti-KS	-	-
Anti-Zo	-	-
Anti-Ha	-	-

**Table 3:** Comparison of existing and proposed classification criteria for ASyS and IIM, adapted from Opinc, 2021 [3] and Marco, 2020 [36].

Solomon et al., 2011 [30]	Connors et al., 2010 [35]	EULAR/ACR 2017 [34]
Antisynthetase antibody plus two major criteria or one major and two minor criteria:	Antisynthetase antibody plus one or more of the following:	Definite IIM: score of at least 7.5 (8.7 with muscle biopsy) Probable IIM: score of at least 5.5 (6.7 with muscle biopsy)
Major Criteria Myositis by Bohan and Peter [33] ILD Minor Criteria Arthritis Raynaud’s phenomenon Mechanic’s hands	Myositis by Bohan and Peter [33] ILD Arthritis Raynaud’s phenomenon Mechanic’s hands Persistent unexplained fever	Anti-Jo-1 Objective symmetrical weakness of upper and lower proximal extremities Proximal leg muscles weaker than distal Dysphagia or esophageal motility disorders Skin lesions (heliotrope rash, Gottron’s papules, and Gottron’s sign) Age of onset Typical features in muscle biopsy and elevated skeletal muscle enzymes

**Table 4:** Clinical features of patients with ASyS by ASAb; Shi, 2017 [41].

Variable	All Patients n = 124	Jo-1 n = 62	PL7 n = 31	PL12 n = 12	EJ n = 19
Mean age of onset (years)	50.4	49.9	51.7	54.0	47.6
Female	75.8%	71.0%	80.6%	75.0%	84.2%
Muscle weakness	63.7%	62.9%	71.0%	66.7%	52.6%
ILD	94.4%	96.8%	83.9%	100%	100%
Rapid onset ILD	8.9%	4.8%	19.4%	8.3%	5.8%
Raynaud’s phenomenon	9.7%	12.9%	3.2%	16.7%	5.3%
Mechanic’s hands	46.0%	46.8%	45.2%	41.7%	47.4%
Arthritis/arthralgia	54.0%	71.0%	41.9%	41.7%	26.3%
Dysphagia	11.3%	8.1%	19.4%	8.3%	10.5%
Malignancy	6.5%	6.5%	6.5%	8.3%	5.3%
*Ro52+*	*29.0%*	*9.7%*	*38.7%*	*66.7%*	*52.6%*

**Table 5:** Prevalence of myositis in ASyS by autoantibody, Cavagna 2019 (n = 828) [14].

Antisynthetase Autoantibody	Myositis (%)	Classic Onset	Hypomyopathic Onset
Anti-Jo-1 (n = 593)	82.1%	80.0%	20.0%
Anti-PL7 (n = 95)	80.0%	69.3%	30.7%
Anti-PL12 (n = 84)	51.2%	72.1%	27.9%
Anti-EJ (n = 38)	84.2%	81.3%	18.7%
Anti-OJ (n = 18)	77.8%	64.3%	35.7%

**Table 6:** Prevalence of ILD in ASyS by autoantibody.

A. Teel, 2022 [48]					
All Antisynthetase Antibodies (n = 994)	Jo-1 n = 712	PL7 n = 78	PL12 n = 23	EJ n = 52	OJ n = 4
93%	83%	87%	91%	98%	100%
**B. Cavagna, 2019 [14]**					
**Antisynthetase Autoantibody** **(n = 828)**	**ILD (%)**	**Acute Onset**	**Chronic Onset**		**Asymptomatic**
Anti-Jo-1 (n = 593)	82.0%	37.3%	41.9%		20.8%
Anti-PL7 (n = 95)	76.8%	38.9%	44.4%		12.6%
Anti-PL12 (n = 84)	83.3%	48.6%	40.0%		11.4%
Anti-EJ (n = 38)	89.5%	63.6%	33.3%		3.0%
Anti-OJ (n = 18)	61.1%	36.4%	54.6%		9.1%

**Table 7:** ASAbs associated with patterns of ILD.

A. de Souza, 2023 [68]						
Antisynthetase Antibody	Frequency in Myositis	Frequency of ILD	NSIP Pattern	UIP Pattern	OP Pattern	DAD Pattern
Jo-1	15–30%	70–80%	10%	25%	5%	50%
PL7	5–10%	84%	25%	50%	50%	-
PL12	<5%	90%	17%	67%	17%	-
KS	1%	90%	62%	27–35%	-	-
OJ	1%	8%	-	-	-	-
EJ	1%	>90%	75%	4%	21%	-
Ha	<1%	60–100%	-	21%	-	-
Zo	<1%	4–38%	60%	10%	30%	-
**B. Jiang, 2021 [69]**						
**Autoantibody n = 84 (n)**	**NSIP**	**OP**	**NSIP + OP**	**UIP**	**Unclassifiable**	
Jo-1 (36)	44.5%	27.8%	19.4%	0%	8.3%	
PL7 (22)	50.0%	18.2%	22.7%	9.1%	0%	
PL12 (9)	22.2%	33.3%	22.2%	11.1%	11.1%	
EJ (12)	58.3%	33.3%	8.3%	0%	0%	
OJ (5)	20.0%	0%	20.0%	40.0%	20.0%	

**Table 9:** The 2023 American College of Rheumatology (ACR) guideline for treatment of ILD in people with systemic autoimmune rheumatic diseases; available on the ACR website.

Treatment Guideline for Myositis Associated ILD	
**Initial Treatment of ILD** **Preferred**	**Mycophenolate Mofetil** **Azathioprine** **Rituximab** **Calcineurin Inhibitors**
Additional options	JAK inhibitors Cyclophosphamide
	Glucocorticoids, short term
Treatment for patients with progressive ILD on initial treatment	IVIG Nintedanib

## Data Availability

Not applicable.

## References

[1] Wells M., Alawi S., Thin K.Y.M., Gunawardena H., Brown A.R., Edey A., Pauling J.D., Barratt S.L., Adamali H.I. (2022). A multidisciplinary approach to the diagnosis of antisynthetase syndrome. Front. Med..

[2] Witt L.J., Curran J.J., Strek M.E. (2016). The Diagnosis and Treatment of Antisynthetase Syndrome. Clin. Pulm. Med..

[3] Opinc A.H., Makowska J.S. (2021). Antisynthetase syndrome—Much more than just a myopathy. Semin. Arthritis Rheum..

[4] Zanframundo G., Faghihi-Kashani S., Scirè C.A., Bonella F., Corte T.J., Doyle T.J., Fiorentino D., Gonzalez-Gay M.A., Hudson M., Kuwana M. (2022). Defining anti-synthetase syndrome: A systematic literature review. Clin. Exp. Rheumatol..

[5] Galindo-Feria A.S., Notarnicola A., Lundberg I.E., Horuluoglu B. (2022). Aminoacyl-tRNA Synthetases: On Anti-Synthetase Syndrome and Beyond. Front. Immunol..

[6] Bunn C.C., Bernstein R.M., Mathews M.B. (1986). Autoantibodies against alanyl-tRNA synthetase and tRNAAla coexist and are associated with myositis. J. Exp. Med..

[7] Targoff I.N. (1990). Autoantibodies to aminoacyl-transfer RNA synthetases for isoleucine and glycine. Two additional synthetases are antigenic in myositis. J. Immunol..

[8] Hirakata M., Suwa A., Nagai S., Kron M.A., Trieu E.P., Mimori T., Akizuki M., Targoff I.N. (1999). Anti-KS: Identification of autoantibodies to asparaginyl-transfer RNA synthetase associated with interstitial lung disease. J. Immunol..

[9] Betteridge Z., Gunawardena H., North J., Slinn J., McHugh N. (2007). Anti-synthetase syndrome: A new autoantibody to phenylalanyl transfer RNA synthetase (anti-Zo) associated with polymyositis and interstitial pneumonia. Rheumatology.

[10] Mimori T., Imura Y., Nakashima R., Yoshifuji H. (2007). Autoantibodies in idiopathic inflammatory myopathy: An update on clinical and pathophysiological significance. Curr. Opin. Rheumatol..

[11] Hervier B., Benveniste O. (2013). Clinical heterogeneity and outcomes of antisynthetase syndrome. Curr. Rheumatol. Rep..

[12] Huang K., Aggarwal R. (2020). Antisynthetase syndrome: A distinct disease spectrum. J. Scleroderma Relat. Disord..

[13] Zhao N., Jiang W., Wu H., Wang P., Wang X., Bai Y., Li Y., Tang Y., Liu Y. (2022). Clinical features, prognostic factors, and survival of patients with antisynthetase syndrome and interstitial lung disease. Front. Immunol..

[14] Cavagna L., Trallero-Araguás E., Meloni F., Cavazzana I., Rojas-Serrano J., Feist E., Zanframundo G., Morandi V., Meyer A., Pereira da Silva J.A. (2019). Influence of Antisynthetase Antibodies Specificities on Antisynthetase Syndrome Clinical Spectrum Time Course. J. Clin. Med..

[15] Darrah E., Rosen A. (2010). Granzyme B cleavage of autoantigens in autoimmunity. Cell Death Differ..

[16] Howard O.M., Dong H.F., Yang D., Raben N., Nagaraju K., Rosen A., Casciola-Rosen L., Härtlein M., Kron M., Yiadom K. (2002). Histidyl-tRNA synthetase and asparaginyl-tRNA synthetase, autoantigens in myositis, activate chemokine receptors on T lymphocytes and immature dendritic cells. J. Exp. Med..

[17] Galindo-Feria A.S., Albrecht I., Fernandes-Cerqueira C., Notarnicola A., James E.A., Herrath J., Dastmalchi M., Sandalova T., Rönnblom L., Jakobsson P.J. (2020). Proinflammatory Histidyl-Transfer RNA Synthetase-Specific CD4+ T Cells in the Blood and Lungs of Patients With Idiopathic Inflammatory Myopathies. Arthritis Rheumatol..

[18] Hervier B., Perez M., Allenbach Y., Devilliers H., Cohen F., Uzunhan Y., Ouakrim H., Dorgham K., Méritet J.F., Longchampt E. (2016). Involvement of NK Cells and NKp30 Pathway in Antisynthetase Syndrome. J. Immunol..

[19] Behrens Pinto G.L., Carboni R.C.S., de Souza F.H.C., Shinjo S.K. (2020). A prospective cross-sectional study of serum IL-17A in antisynthetase syndrome. Clin. Rheumatol..

[20] Mitsdoerffer M., Lee Y., Jäger A., Kim H.J., Korn T., Kolls J.K., Cantor H., Bettelli E., Kuchroo V.K. (2010). Proinflammatory T helper type 17 cells are effective B-cell helpers. Proc. Natl. Acad. Sci. USA.

[21] Roşu A., Mărgăritescu C., Stepan A., Muşetescu A., Ene M. (2012). IL-17 patterns in synovium, serum and synovial fluid from treatment-naïve, early rheumatoid arthritis patients. Rom. J. Morphol. Embryol..

[22] Chen X.Q., Yu Y.C., Deng H.H., Sun J.Z., Dai Z., Wu Y.W., Yang M. (2010). Plasma IL-17A is increased in new-onset SLE patients and associated with disease activity. J. Clin. Immunol..

[23] Chen T., Li Y., Sun R., Hu H., Liu Y., Herrmann M., Zhao Y., Muñoz L.E. (2021). Receptor-Mediated NETosis on Neutrophils. Front. Immunol..

[24] Khandpur R., Carmona-Rivera C., Vivekanandan-Giri A., Gizinski A., Yalavarthi S., Knight J.S., Friday S., Li S., Patel R.M., Subramanian V. (2013). NETs are a source of citrullinated autoantigens and stimulate inflammatory responses in rheumatoid arthritis. Sci. Transl. Med..

[25] Seto N., Torres-Ruiz J.J., Carmona-Rivera C., Pinal-Fernandez I., Pak K., Purmalek M.M., Hosono Y., Fernandes-Cerqueira C., Gowda P., Arnett N. (2020). Neutrophil dysregulation is pathogenic in idiopathic inflammatory myopathies. JCI Insight.

[26] López-Mejías R., Remuzgo-Martínez S., Genre F., Pulito-Cueto V., Rozas S.M.F., Llorca J., Fernández D.I., Cuesta V.M.M., Ortego-Centeno N., Gómez N.P. (2020). Influence of MUC5B gene on antisynthetase syndrome. Sci. Rep..

[27] Remuzgo-Martínez S., Atienza-Mateo B., Ocejo-Vinyals J.G., Genre F., Pulito-Cueto V., Mora-Cuesta V.M., Iturbe-Fernández D., Lera-Gómez L., Pérez-Fernández R., Prieto-Peña D. (2021). Role of MUC1 rs4072037 polymorphism and serum KL-6 levels in patients with antisynthetase syndrome. Sci. Rep..

[28] Ponce-Gallegos M.A., Ramos-Martínez E., García-Carmona A., Mejía M., Nava-Quiroz K.J., Pérez-Rubio G., Ambrocio-Ortiz E., González-Pérez M.I., Buendía-Roldán I., Rojas-Serrano J. (2020). Genetic Susceptibility to Antisynthetase Syndrome Associated With Single-Nucleotide Variants in the IL1B Gene That Lead Variation in IL-1β Serum Levels. Front. Med..

[29] Pinal-Fernandez I., Casal-Dominguez M., Huapaya J.A., Albayda J., Paik J.J., Johnson C., Silhan L., Christopher-Stine L., Mammen A.L., Danoff S.K. (2017). A longitudinal cohort study of the anti-synthetase syndrome: Increased severity of interstitial lung disease in black patients and patients with anti-PL7 and anti-PL12 autoantibodies. Rheumatology.

[30] Solomon J., Swigris J.J., Brown K.K. (2011). Myositis-related interstitial lung disease and antisynthetase syndrome. J. Bras. Pneumol..

[31] Lilleker J.B., Vencovsky J., Wang G., Wedderburn L.R., Diederichsen L.P., Schmidt J., Oakley P., Benveniste O., Danieli M.G., Danko K. (2018). The EuroMyositis registry: An international collaborative tool to facilitate myositis research. Ann. Rheum. Dis..

[32] Noguchi E., Uruha A., Suzuki S., Hamanaka K., Ohnuki Y., Tsugawa J., Watanabe Y., Nakahara J., Shiina T., Suzuki N. (2017). Skeletal Muscle Involvement in Antisynthetase Syndrome. JAMA Neurol..

[33] Bohan A., Peter J.B. (1975). Polymyositis and dermatomyositis (first of two parts). N. Engl. J. Med..

[34] Lundberg I.E., Tjärnlund A., Bottai M., Werth V.P., Pilkington C., de Visser M., Alfredsson L., Amato A.A., Barohn R.J., Liang M.H. (2017). 2017 European League Against Rheumatism/American College of Rheumatology Classification Criteria for Adult and Juvenile Idiopathic Inflammatory Myopathies and Their Major Subgroups. Arthritis Rheumatol..

[35] Connors G.R., Christopher-Stine L., Oddis C.V., Danoff S.K. (2010). Interstitial lung disease associated with the idiopathic inflammatory myopathies: What progress has been made in the past 35 years?. Chest.

[36] Marco J.L., Collins B.F. (2020). Clinical manifestations and treatment of antisynthetase syndrome. Best. Pract. Res. Clin. Rheumatol..

[37] Cavagna L., Nuño L., Scirè C.A., Govoni M., Longo F.J.L., Franceschini F., Neri R., Castañeda S., Giraldo W.A.S., Caporali R. (2015). Clinical Spectrum Time Course in Anti Jo-1 Positive Antisynthetase Syndrome: Results From an International Retrospective Multicenter Study. Medicine.

[38] Cavagna L., Nuño L., Scirè C.A., Govoni M., Longo F.J., Franceschini F., Neri R., Castañeda S., Sifuentes Giraldo W.A., Caporali R. (2017). Serum Jo-1 Autoantibody and Isolated Arthritis in the Antisynthetase Syndrome: Review of the Literature and Report of the Experience of AENEAS Collaborative Group. Clin. Rev. Allergy Immunol..

[39] González-Gay M.A., Montecucco C., Selva-O’Callaghan A., Trallero-Araguas E., Molberg O., Andersson H., Rojas-Serrano J., Perez-Roman D.I., Bauhammer J., Fiehn C. (2018). Timing of onset affects arthritis presentation pattern in antisyntethase syndrome. Clin. Exp. Rheumatol..

[40] Baccaro A.C.C.D., Behrens Pinto G.L., Carboni R.C.S., Shinjo S.K. (2020). The clinical manifestations at the onset of antisynthetase syndrome: A chameleon with multiple faces. Reumatismo.

[41] Shi J., Li S., Yang H., Zhang Y., Peng Q., Lu X., Wang G. (2017). Clinical Profiles and Prognosis of Patients with Distinct Antisynthetase Autoantibodies. J. Rheumatol..

[42] Hervier B., Devilliers H., Stanciu R., Meyer A., Uzunhan Y., Masseau A., Dubucquoi S., Hatron P.Y., Musset L., Wallaert B. (2012). Hierarchical cluster and survival analyses of antisynthetase syndrome: Phenotype and outcome are correlated with anti-tRNA synthetase antibody specificity. Autoimmun. Rev..

[43] Marie I., Hatron P.Y., Dominique S., Cherin P., Mouthon L., Menard J.F., Levesque H., Jouen F. (2012). Short-term and long-term outcome of anti-Jo1-positive patients with anti-Ro52 antibody. Semin. Arthritis Rheum..

[44] Marie I., Josse S., Decaux O., Dominique S., Diot E., Landron C., Roblot P., Jouneau S., Hatron P.Y., Tiev K.P. (2012). Comparison of long-term outcome between anti-Jo1- and anti-PL7/PL12 positive patients with antisynthetase syndrome. Autoimmun. Rev..

[45] Andersson H., Kirkhus E., Garen T., Walle-Hansen R., Merckoll E., Molberg Ø. (2017). Comparative analyses of muscle MRI and muscular function in anti-synthetase syndrome patients and matched controls: A cross-sectional study. Arthritis Res. Ther..

[46] McGrath E.R., Doughty C.T., Amato A.A. (2018). Autoimmune Myopathies: Updates on Evaluation and Treatment. Neurotherapeutics.

[47] Aggarwal R., Cassidy E., Fertig N., Koontz D.C., Lucas M., Ascherman D.P., Oddis C.V. (2014). Patients with non-Jo-1 anti-tRNA-synthetase autoantibodies have worse survival than Jo-1 positive patients. Ann. Rheum. Dis..

[48] Teel A., Lu J., Park J., Singh N., Basharat P. (2022). The Role of Myositis-Specific Autoantibodies and the Management of Interstitial Lung Disease in Idiopathic Inflammatory Myopathies: A Systematic Review. Semin. Arthritis Rheum..

[49] Zamora A.C., Hoskote S.S., Abascal-Bolado B., White D., Cox C.W., Ryu J.H., Moua T. (2016). Clinical features and outcomes of interstitial lung disease in anti-Jo-1 positive antisynthetase syndrome. Respir. Med..

[50] Waseda Y., Johkoh T., Egashira R., Sumikawa H., Saeki K., Watanabe S., Matsunuma R., Takato H., Ichikawa Y., Hamaguchi Y. (2016). Antisynthetase syndrome: Pulmonary computed tomography findings of adult patients with antibodies to aminoacyl-tRNA synthetases. Eur. J. Radiol..

[51] Debray M.P., Borie R., Revel M.P., Naccache J.M., Khalil A., Toper C., Israel-Biet D., Estellat C., Brillet P.Y. (2015). Interstitial lung disease in anti-synthetase syndrome: Initial and follow-up CT findings. Eur. J. Radiol..

[52] Yamakawa H., Hagiwara E., Kitamura H., Iwasawa T., Otoshi R., Aiko N., Katano T., Shintani R., Ikeda S., Okuda R. (2018). Predictive Factors for the Long-Term Deterioration of Pulmonary Function in Interstitial Lung Disease Associated with Anti-Aminoacyl-tRNA Synthetase Antibodies. Respiration.

[53] Chartrand S., Swigris J.J., Peykova L., Chung J., Fischer A. (2016). A Multidisciplinary Evaluation Helps Identify the Antisynthetase Syndrome in Patients Presenting as Idiopathic Interstitial Pneumonia. J. Rheumatol..

[54] Chatterjee S., Prayson R., Farver C. (2013). Antisynthetase syndrome: Not just an inflammatory myopathy. Cleve Clin. J. Med..

[55] Hervier B., Meyer A., Dieval C., Uzunhan Y., Devilliers H., Launay D., Canuet M., Têtu L., Agard C., Sibilia J. (2013). Pulmonary hypertension in antisynthetase syndrome: Prevalence, aetiology and survival. Eur. Respir. J..

[56] Rojas-Serrano J., Herrera-Bringas D., Mejía M., Rivero H., Mateos-Toledo H., Figueroa J.E. (2015). Prognostic factors in a cohort of antisynthetase syndrome (ASS): Serologic profile is associated with mortality in patients with interstitial lung disease (ILD). Clin. Rheumatol..

[57] Bachmeyer C., Tillie-Leblond I., Lacert A., Cadranel J., Aractingi S. (2007). “Mechanic’s hands”: A misleading cutaneous sign of the antisynthetase syndrome. Br. J. Dermatol..

[58] Cox J.T., Gullotti D.M., Mecoli C.A., Lahouti A.H., Albayda J., Paik J., Johnson C., Danoff S.K., Mammen A.L., Christopher-Stine L. (2017). “Hiker’s feet”: A novel cutaneous finding in the inflammatory myopathies. Clin. Rheumatol..

[59] Wernham M., Montague S.J. (2017). Mechanic’s hands and hiker’s feet in antisynthetase syndrome. CMAJ.

[60] Sebastiani M., Triantafyllias K., Manfredi A., González-Gay M.A., Palmou-Fontana N., Cassone G., Drott U., Delbrück C., Rojas-Serrano J., Bertolazzi C. (2019). Nailfold Capillaroscopy Characteristics of Antisynthetase Syndrome and Possible Clinical Associations: Results of a Multicenter International Study. J. Rheumatol..

[61] Yoshida A., Gono T., Okazaki Y., Shirai Y., Takeno M., Kuwana M. (2022). Severe digital ischemia as an unrecognized manifestation in patients with antisynthetase autoantibodies: Case series and systematic literature review. J. Scleroderma Relat. Disord..

[62] Dieval C., Deligny C., Meyer A., Cluzel P., Champtiaux N., Lefevre G., Saadoun D., Sibilia J., Pellegrin J.L., Hachulla E. (2015). Myocarditis in Patients With Antisynthetase Syndrome: Prevalence, Presentation, and Outcomes. Medicine.

[63] Labirua-Iturburu A., Selva-O’Callaghan A., Vincze M., Dankó K., Vencovsky J., Fisher B., Charles P., Dastmalchi M., Lundberg I.E. (2012). Anti-PL-7 (anti-threonyl-tRNA synthetase) antisynthetase syndrome: Clinical manifestations in a series of patients from a European multicenter study (EUMYONET) and review of the literature. Medicine.

[64] Sawal N., Mukhopadhyay S., Rayancha S., Moore A., Garcha P., Kumar A., Kaul V. (2021). A narrative review of interstitial lung disease in anti-synthetase syndrome: A clinical approach. J. Thorac. Dis..

[65] Baratella E., Marrocchio C., Cifaldi R., Santagiuliana M., Bozzato A.M., Crivelli P., Ruaro B., Salton F., Confalonieri M., Cova M.A. (2021). Interstitial lung disease in patients with antisynthetase syndrome: A retrospective case series study. Jpn. J. Radiol..

[66] Gomes Ferreira S., Fernandes L., Santos S., Ferreira S., Teixeira M. (2024). From Suspected COVID-19 to Anti-synthetase Syndrome: A Diagnostic Challenge in the Pandemic Era. Cureus.

[67] Alfraji N., Mazahir U., Chaudhri M., Miskoff J. (2021). Anti-synthetase syndrome: A rare and challenging diagnosis for bilateral ground-glass opacities-a case report with literature review. BMC Pulm. Med..

[68] De Souza F.H.C., De Araújo D.B., Hoff L.S., Baldi B.G., Faria M.S.M.S., Da Rocha Junior L.F., Da Silva L.R.S., Behrens Pinto G.L., Bezerra M.C., Miossi R. (2023). Diagnosis and treatment of interstitial lung disease related to systemic autoimmune myopathies: A narrative review. Reumatismo.

[69] Jiang M., Dong X., Zheng Y. (2021). Clinical characteristics of interstitial lung diseases positive to different anti-synthetase antibodies. Medicine.

[70] Hallowell R.W., Danoff S.K. (2023). Diagnosis and Management of Myositis-Associated Lung Disease. Chest.

[71] da Silva L.M.B., Borges I.B.P., Shinjo S.K. (2023). High prevalence of necrotising myopathy pattern in muscle biopsies of patients with anti-Jo-1 antisynthetase syndrome. Clin. Exp. Rheumatol..

[72] Tanboon J., Inoue M., Hirakawa S., Tachimori H., Hayashi S., Noguchi S., Okiyama N., Fujimoto M., Suzuki S., Nishino I. (2023). Muscle pathology of antisynthetase syndrome according to antibody subtypes. Brain Pathol..

[73] Hamaguchi Y., Fujimoto M., Matsushita T., Kaji K., Komura K., Hasegawa M., Kodera M., Muroi E., Fujikawa K., Seishima M. (2013). Common and distinct clinical features in adult patients with anti-aminoacyl-tRNA synthetase antibodies: Heterogeneity within the syndrome. PLoS ONE.

[74] Sreevilasan S.K., Devarasetti P., Narahari N.K., Desai A., Rajasekhar L. (2021). Clinical profile and treatment outcomes in antisynthetase syndrome: A tertiary centre experience. Rheumatol. Adv. Pract..

[75] Lefèvre G., Meyer A., Launay D., Machelart I., DeBandt M., Michaud J., Tournadre A., Godmer P., Kahn J.E., Behra-Marsac A. (2015). Seronegative polyarthritis revealing antisynthetase syndrome: A multicentre study of 40 patients. Rheumatology.

[76] Marie I., Josse S., Hatron P.Y., Dominique S., Hachulla E., Janvresse A., Cherin P., Mouthon L., Vittecoq O., Menard J.F. (2013). Interstitial lung disease in anti-Jo-1 patients with antisynthetase syndrome. Arthritis Care Res..

[77] Arcani R., Rey L., Mazziotto A., Bertin D., Kaplanski G., Jarrot P.A., Lafforgue P., Venton G., Heim X., Villani P. (2023). Anti-Jo-1 autoantibodies: Biomarkers of severity and evolution of the disease in antisynthetase syndrome. Arthritis Res. Ther..

[78] Hervier B., Uzunhan Y., Hachulla E., Benveniste O., Nunes H., Delaval P., Musset L., Dubucquoi S., Wallaert B., Hamidou M. (2011). Antisynthetase syndrome positive for anti-threonyl-tRNA synthetase (anti-PL7) antibodies. Eur. Respir. J..

[79] Zhan X., Yan W., Wang Y., Li Q., Shi X., Gao Y., Ye Q. (2021). Clinical features of anti-synthetase syndrome associated interstitial lung disease: A retrospective cohort in China. BMC Pulm. Med..

[80] Abel A., Lazaro E., Ralazamahaleo M., Pierrisnard E., Suzon B., Bonnet F., Mercié P., Macey J., Agossou M., Viallard J.F. (2023). Phenotypic Profiles Among 72 Caucasian and Afro-Caribbean Patients with Antisynthetase Syndrome Involving Anti-PL7 or Anti-PL12 Autoantibodies. Eur. J. Intern. Med..

[81] Vulsteke J.B., Satoh M., Malyavantham K., Bossuyt X., De Langhe E., Mahler M. (2019). Anti-OJ autoantibodies: Rare or underdetected?. Autoimmun. Rev..

[82] Liu Y., Liu X., Xie M., Chen Z., He J., Wang Z., Dai J., Cai H. (2020). Clinical characteristics of patients with anti-EJ antisynthetase syndrome associated interstitial lung disease and literature review. Respir. Med..

[83] Okayasu K., Ohtani Y., Takemura T., Uchibori K., Tamaoka M., Furuiye M., Miyazaki Y., Inase N., Yoshizawa Y. (2009). Nonspecific interstitial pneumonia (NSIP) associated with anti-KS antibody: Differentiation from idiopathic NSIP. Intern. Med..

[84] Schneider F., Aggarwal R., Bi D., Gibson K., Oddis C., Yousem S.A. (2015). The pulmonary histopathology of anti-KS transfer RNA synthetase syndrome. Arch. Pathol. Lab. Med..

[85] Tansley S.L., Betteridge Z., Lu H., Davies E., Rothwell S., New P.P., Chinoy H., Gordon P., Gunawardena H., Lloyd M. (2020). The myositis clinical phenotype associated with anti-Zo autoantibodies: A case series of nine UK patients. Rheumatology.

[86] Ueda-Hayakawa I., Hasegawa M., Kumada S., Tanaka C., Komura K., Hamaguchi Y., Takehara K., Fujimoto M. (2010). Usefulness of anti-cyclic citrullinated peptide antibody and rheumatoid factor to detect rheumatoid arthritis in patients with systemic sclerosis. Rheumatology.

[87] Pakozdi A., Nihtyanova S., Moinzadeh P., Ong V.H., Black C.M., Denton C.P. (2011). Clinical and serological hallmarks of systemic sclerosis overlap syndromes. J. Rheumatol..

[88] Hoffman I.E., Peene I., Cebecauer L., Isenberg D., Huizinga T.W., Union A., Meheus L., De Bosschere K., Hulstaert F., Veys E.M. (2005). Presence of rheumatoid factor and antibodies to citrullinated peptides in systemic lupus erythematosus. Ann. Rheum. Dis..

[89] Qing Y.F., Zhang Q.B., Zhou J.G., Yuan G.H., Wei J., Xing Y., Liu J.P., Jiang L., Chen J.P. (2009). The detecting and clinical value of anti-cyclic citrullinated peptide antibodies in patients with systemic lupus erythematosus. Lupus.

[90] Kakumanu P., Sobel E.S., Narain S., Li Y., Akaogi J., Yamasaki Y., Segal M.S., Hahn P.C., Chan E.K., Reeves W.H. (2009). Citrulline dependence of anti-cyclic citrullinated peptide antibodies in systemic lupus erythematosus as a marker of deforming/erosive arthritis. J. Rheumatol..

[91] Meyer A., Lefevre G., Bierry G., Duval A., Ottaviani S., Meyer O., Tournadre A., Le Goff B., Messer L., Buchdahl A.L. (2015). In antisynthetase syndrome, ACPA are associated with severe and erosive arthritis: An overlapping rheumatoid arthritis and antisynthetase syndrome. Medicine.

[92] Bauhammer J., Blank N., Max R., Lorenz H.M., Wagner U., Krause D., Fiehn C. (2016). Rituximab in the Treatment of Jo1 Antibody-associated Antisynthetase Syndrome: Anti-Ro52 Positivity as a Marker for Severity and Treatment Response. J. Rheumatol..

[93] Vojinovic T., Cavazzana I., Ceruti P., Fredi M., Modina D., Berlendis M., Franceschini F. (2021). Predictive Features and Clinical Presentation of Interstitial Lung Disease in Inflammatory Myositis. Clin. Rev. Allergy Immunol..

[94] Shao C., Sun Y., Huang H., Zhang Z., Pan R., Xu K., Zhang X., Zhang Y., Xu Z. (2022). Myositis specific antibodies are associated with isolated anti-Ro-52 associated interstitial lung disease. Rheumatology.

[95] Chaudhry S., Christopher-Stine L. (2023). Myositis interstitial lung disease and autoantibodies. Front. Med..

[96] Pepper E., Vilar L., Ward I.M. (2023). Clinical Characteristics and Prognostic Value of Ro52/SSA Antibodies in Idiopathic Inflammatory Myopathies. J. Clin. Rheumatol..

[97] Limaye V.S., Cassidy J., Scott G., Roberts-Thomson P.J., Gillis D. (2008). Anti-Ro52 antibodies, antisynthetase antibodies, and antisynthetase syndrome. Clin. Rheumatol..

[98] Cruellas M.G., Viana V.D.S.T., Levy-Neto M., Souza F.H., Shinjo S.K. (2013). Myositis-specific and myositis-associated autoantibody profiles and their clinical associations in a large series of patients with polymyositis and dermatomyositis. Clinics.

[99] Yamasaki Y., Satoh M., Mizushima M., Okazaki T., Nagafuchi H., Ooka S., Shibata T., Nakano H., Ogawa H., Azuma K. (2016). Clinical subsets associated with different anti-aminoacyl transfer RNA synthetase antibodies and their association with coexisting anti-Ro52. Mod. Rheumatol..

[100] Bozzalla-Cassione E., Zanframundo G., Biglia A., Bellis E., Bozzini S., Codullo V., Vertui V., Alpini C., Valentini A., Preda L. (2022). Anti-Ro52 antibodies positivity in antisynthetase syndrome: A single centre cohort study. Clin. Exp. Rheumatol..

[101] La Corte R., Lo Mo Naco A., Locaputo A., Dolzani F., Trotta F. (2006). In patients with antisynthetase syndrome the occurrence of anti-Ro/SSA antibodies causes a more severe interstitial lung disease. Autoimmunity.

[102] Váncsa A., Csípo I., Németh J., Dévényi K., Gergely L., Dankó K. (2009). Characteristics of interstitial lung disease in SS-A positive/Jo-1 positive inflammatory myopathy patients. Rheumatol. Int..

[103] Chen H., Liu H., Lyu W., Liu Y., Huang M., Zhang Y., Qiu Y., Xiao Y., Cai H., Dai J. (2023). An observational study of clinical recurrence in patients with interstitial lung disease related to the antisynthetase syndrome. Clin. Rheumatol..

[104] Swigris J.J., Olson A.L., Fischer A., Lynch D.A., Cosgrove G.P., Frankel S.K., Meehan R.T., Brown K.K. (2006). Mycophenolate mofetil is safe, well tolerated, and preserves lung function in patients with connective tissue disease-related interstitial lung disease. Chest.

[105] Saketkoo L.A., Espinoza L.R. (2009). Experience of mycophenolate mofetil in 10 patients with autoimmune-related interstitial lung disease demonstrates promising effects. Am. J. Med. Sci..

[106] Morganroth P.A., Kreider M.E., Werth V.P. (2010). Mycophenolate mofetil for interstitial lung disease in dermatomyositis. Arthritis Care Res..

[107] Fischer A., Brown K.K., Du Bois R.M., Frankel S.K., Cosgrove G.P., Fernandez-Perez E.R., Huie T.J., Krishnamoorthy M., Meehan R.T., Olson A.L. (2013). Mycophenolate mofetil improves lung function in connective tissue disease-associated interstitial lung disease. J. Rheumatol..

[108] Tsuchiya H., Tsuno H., Inoue M., Takahashi Y., Yamashita H., Kaneko H., Kano T., Mimori A. (2014). Mycophenolate mofetil therapy for rapidly progressive interstitial lung disease in a patient with clinically amyopathic dermatomyositis. Mod. Rheumatol..

[109] Mira-Avendano I.C., Parambil J.G., Yadav R., Arrossi V., Xu M., Chapman J.T., Culver D.A. (2013). A retrospective review of clinical features and treatment outcomes in steroid-resistant interstitial lung disease from polymyositis/dermatomyositis. Respir. Med..

[110] Huapaya J.A., Silhan L., Pinal-Fernandez I., Casal-Dominguez M., Johnson C., Albayda J., Paik J.J., Sanyal A., Mammen A.L., Christopher-Stine L. (2019). Long-Term Treatment With Azathioprine and Mycophenolate Mofetil for Myositis-Related Interstitial Lung Disease. Chest.

[111] Ge Y., Zhou H., Shi J., Ye B., Peng Q., Lu X., Wang G. (2015). The efficacy of tacrolimus in patients with refractory dermatomyositis/polymyositis: A systematic review. Clin. Rheumatol..

[112] Hozumi H., Fujisawa T., Nakashima R., Yasui H., Suzuki Y., Kono M., Karayama M., Furuhashi K., Enomoto N., Inui N. (2019). Efficacy of Glucocorticoids and Calcineurin Inhibitors for Anti-aminoacyl-tRNA Synthetase Antibody-positive Polymyositis/dermatomyositis-associated Interstitial Lung Disease: A Propensity Score-matched Analysis. J. Rheumatol..

[113] Lee D.S.W., Rojas O.L., Gommerman J.L. (2021). B cell depletion therapies in autoimmune disease: Advances and mechanistic insights. Nat. Rev. Drug Discov..

[114] Sem M., Molberg O., Lund M.B., Gran J.T. (2009). Rituximab treatment of the anti-synthetase syndrome: A retrospective case series. Rheumatology.

[115] Marie I., Dominique S., Janvresse A., Levesque H., Menard J.F. (2012). Rituximab therapy for refractory interstitial lung disease related to antisynthetase syndrome. Respir. Med..

[116] Andersson H., Sem M., Lund M.B., Aaløkken T.M., Günther A., Walle-Hansen R., Garen T., Molberg Ø. (2015). Long-term experience with rituximab in anti-synthetase syndrome-related interstitial lung disease. Rheumatology.

[117] Doyle T.J., Dhillon N., Madan R., Cabral F., Fletcher E.A., Koontz D.C., Aggarwal R., Osorio J.C., Rosas I.O., Oddis C.V. (2018). Rituximab in the Treatment of Interstitial Lung Disease Associated with Antisynthetase Syndrome: A Multicenter Retrospective Case Review. J. Rheumatol..

[118] Allenbach Y., Guiguet M., Rigolet A., Marie I., Hachulla E., Drouot L., Jouen F., Jacquot S., Mariampillai K., Musset L. (2015). Efficacy of Rituximab in Refractory Inflammatory Myopathies Associated with Anti- Synthetase Auto-Antibodies: An Open-Label, Phase II Trial. PLoS ONE.

[119] Ge Y., Peng Q., Zhang S., Zhou H., Lu X., Wang G. (2015). Cyclophosphamide treatment for idiopathic inflammatory myopathies and related interstitial lung disease: A systematic review. Clin. Rheumatol..

[120] Barba T., Fort R., Cottin V., Provencher S., Durieu I., Jardel S., Hot A., Reynaud Q., Lega J.C. (2019). Treatment of idiopathic inflammatory myositis associated interstitial lung disease: A systematic review and meta-analysis. Autoimmun. Rev..

[121] Moreno-Torres V., Martín-Iglesias D., Vivero F., González-Echavarri C., García-Moyano M., Enghelmayer J.I., Malfante P., Gaser A., Ruiz-Irastorza G., EPIMAR cohort Investigators (2023). Intravenous cyclophosphamide improves functional outcomes in interstitial lung disease related to idiopathic inflammatory myopathies. Semin. Arthritis Rheum..

[122] Marie I., Hatron P.Y., Cherin P., Hachulla E., Diot E., Vittecoq O., Menard J.F., Jouen F., Dominique S. (2013). Functional outcome and prognostic factors in anti-Jo1 patients with antisynthetase syndrome. Arthritis Res. Ther..

[123] McPherson M., Economidou S., Liampas A., Zis P., Parperis K. (2022). Management of MDA-5 antibody positive clinically amyopathic dermatomyositis associated interstitial lung disease: A systematic review. Semin. Arthritis Rheum..

[124] Langlois V., Gillibert A., Uzunhan Y., Chabi M.L., Hachulla E., Landon-Cardinal O., Mariampillai K., Champtiaux N., Nunes H., Benveniste O. (2020). Rituximab and Cyclophosphamide in Antisynthetase Syndrome-related Interstitial Lung Disease: An Observational Retrospective Study. J. Rheumatol..

[125] Maher T.M., Tudor V.A., Saunders P., Gibbons M.A., Fletcher S.V., Denton C.P., Hoyles R.K., Parfrey H., Renzoni E.A., Kokosi M. (2023). Rituximab versus intravenous cyclophosphamide in patients with connective tissue disease-associated interstitial lung disease in the UK (RECITAL): A double-blind, double-dummy, randomised, controlled, phase 2b trial. Lancet Respir. Med..

[126] Mougiakakos D., Krönke G., Völkl S., Kretschmann S., Aigner M., Kharboutli S., Böltz S., Manger B., Mackensen A., Schett G. (2021). CD19-Targeted CAR T Cells in Refractory Systemic Lupus Erythematosus. N. Engl. J. Med..

[127] Boulougoura A., Gendelman H., Surmachevska N., Kyttaris V.C. (2023). Journal Club: Anti-CD19 Chimeric Antigen Receptor T Cell Therapy for Refractory Systemic Lupus Erythematosus. ACR Open Rheumatol..

[128] Mackensen A., Müller F., Mougiakakos D., Böltz S., Wilhelm A., Aigner M., Völkl S., Simon D., Kleyer A., Munoz L. (2023). Author Correction: Anti-CD19 CAR T cell therapy for refractory systemic lupus erythematosus. Nat. Med..

[129] Bergmann C., Müller F., Distler J.H.W., Györfi A.H., Völkl S., Aigner M., Kretschmann S., Reimann H., Harrer T., Bayerl N. (2023). Treatment of a patient with severe systemic sclerosis (SSc) using CD19-targeted CAR T cells. Ann. Rheum. Dis..

[130] Müller F., Boeltz S., Knitza J., Aigner M., Völkl S., Kharboutli S., Reimann H., Taubmann J., Kretschmann S., Rösler W. (2023). CD19-targeted CAR T cells in refractory antisynthetase syndrome. Lancet.

[131] Pecher A.C., Hensen L., Klein R., Schairer R., Lutz K., Atar D., Seitz C., Stanger A., Schneider J., Braun C. (2023). CD19-Targeting CAR T Cells for Myositis and Interstitial Lung Disease Associated With Antisynthetase Syndrome. JAMA.

[132] Taubmann J., Knitza J., Müller F., Völkl S., Aigner M., Kleyer A., Gary R., Kretschmann S., Boeltz S., Atzinger A. (2023). Rescue therapy of antisynthetase syndrome with CD19-targeted CAR-T-cells after failure of several B cell depleting antibodies. Rheumatology.

[133] Mueller F., Schwingen N.R., Stabel L., Aigner M., Taubmann J., Kretschmann S., Reimann H., Lutzny-Geier G., Schletter M., Eisenhauer N. (2023). CAR-T Cell Therapy in Patients with Refractory Systemic Autoimmune Diseases Exhibits Less Inflammation, Toxicities and Different Cellular Dynamics Compared to Patients with B Cell Lymphoma. Blood.

[134] Campochiaro C., Farina N., De Luca G., Trignani G., Tomelleri A., Matucci-Cerinic M., Dagna L. (2023). Anakinra for the Treatment of Antisynthetase Syndrome: A Monocentric Case Series and a Systematic Literature Review. J. Rheumatol..

[135] Baumann Benvenuti F., Dudler J. (2023). Long-lasting improvement of refractory antisynthetase syndrome with tocilizumab: A report of two cases. RMD Open.

[136] Xia N., Hong S.M., Zhang X., Shao C., Yan N., Ding H., Guo Q. (2024). Efficacy and safety of abatacept for interstitial lung disease associated with antisynthetase syndrome: A case series. Clin. Exp. Rheumatol..

[137] Pineton de Chambrun M., Hervier B., Chauveau S., Tandjaoui-Lambiotte Y., Combes A., Uzunhan Y. (2020). Tofacitinib in antisynthetase syndrome-related rapidly progressive interstitial lung disease. Rheumatology.

[138] Sugino K., Ono H., Saito M., Ando M., Tsuboi E. (2023). Successful baricitinib treatment of refractory anti-synthetase syndrome associated with interstitial lung disease. Respirol. Case Rep..

[139] Beckett M., Dutz J., Huang K. (2024). Upadacitinib therapy in refractory inflammatory myositis: A case series of 10 patients. RMD Open.

[140] Velikova T., Sekulovski M., Bogdanova S., Vasilev G., Peshevska-Sekulovska M., Miteva D., Georgiev T. (2023). Intravenous Immunoglobulins as Immunomodulators in Autoimmune Diseases and Reproductive Medicine. Antibodies.

[141] Aggarwal R., Charles-Schoeman C., Schessl J., Bata-Csörgő Z., Dimachkie M.M., Griger Z., Moiseev S., Oddis C., Schiopu E., Vencovský J. (2022). Trial of Intravenous Immune Globulin in Dermatomyositis. N. Engl. J. Med..

[142] Huapaya J.A., Hallowell R., Silhan L., Pinal-Fernandez I., Casal-Dominguez M., Johnson C., Albayda J., Paik J.J., Lin C.T., Hussien A. (2019). Long-term treatment with human immunoglobulin for antisynthetase syndrome-associated interstitial lung disease. Respir. Med..

[143] Omotoso B.A., Ogden M.I., Balogun R.A. (2015). Therapeutic plasma exchange in antisynthetase syndrome with severe interstitial lung disease. J. Clin. Apher..

[144] Thompson T.Z., Bobr A., Juskewitch J.E., Winters J.L. (2023). Therapeutic plasma exchange for steroid refractory idiopathic inflammatory myopathies with interstitial lung disease. J. Clin. Apher..

[145] Wells A.U., Flaherty K.R., Brown K.K., Inoue Y., Devaraj A., Richeldi L., Moua T., Crestani B., Wuyts W.A., Stowasser S. (2020). Nintedanib in patients with progressive fibrosing interstitial lung diseases-subgroup analyses by interstitial lung disease diagnosis in the INBUILD trial: A randomised, double-blind, placebo-controlled, parallel-group trial. Lancet Respir. Med..

[146] FDA. https://www.fda.gov/news-events/press-announcements/fda-approves-first-treatment-group-progressive-interstitial-lung-diseases.

[147] Flaherty K.R., Wells A.U., Cottin V., Devaraj A., Walsh S.L.F., Inoue Y., Richeldi L., Kolb M., Tetzlaff K., Stowasser S. (2019). Nintedanib in Progressive Fibrosing Interstitial Lung Diseases. N. Engl. J. Med..

[148] Distler O., Highland K.B., Gahlemann M., Azuma A., Fischer A., Mayes M.D., Raghu G., Sauter W., Girard M., Alves M. (2019). Nintedanib for Systemic Sclerosis-Associated Interstitial Lung Disease. N. Engl. J. Med..

[149] Liang J., Cao H., Yang Y., Ke Y., Yu Y., Sun C., Yue L., Lin J. (2021). Efficacy and Tolerability of Nintedanib in Idiopathic-Inflammatory-Myopathy-Related Interstitial Lung Disease: A Pilot Study. Front. Med..

[150] Wang H., Wang Y., Sun D., Yu S., Du X., Ye Q. (2023). Progressive pulmonary fibrosis in myositis-specific antibody-positive interstitial pneumonia: A retrospective cohort study. Front. Med..

